# Switching from TNFα inhibitor to tacrolimus as maintenance therapy in rheumatoid arthritis after achieving low disease activity with TNFα inhibitors and methotrexate: 24-week result from a non-randomized, prospective, active-controlled trial

**DOI:** 10.1186/s13075-021-02566-z

**Published:** 2021-07-08

**Authors:** Sang Youn Jung, Jung Hee Koh, Ki-Jo Kim, Yong-Wook Park, Hyung-In Yang, Sung Jae Choi, Jisoo Lee, Chan-Bum Choi, Wan-Uk Kim

**Affiliations:** 1 Division of Rheumatology, Department of Internal Medicine, CHA Bundang Medical Center, CHA University, Seongnam, South Korea; 2 Division of Rheumatology, Department of Internal Medicine, Bucheon St. Mary’s Hospital, the Catholic University of Korea, Seoul, South Korea; 3 Division of Rheumatology, Department of Internal Medicine, St. Vincent Hospital, the Catholic University of Korea, Seoul, South Korea; 4 Division of Rheumatology, Department of Internal Medicine, Chonnam National University Medical School and Hospital, Gwangju, South Korea; 5 College of Medicine, Kyung Hee University Hospital at Gangdong, Seoul, South Korea; 6 Division of Rheumatology, Department of Internal Medicine, Korea University Ansan Hospital, Ansan, South Korea; 7 Division of Rheumatology, Department of Internal Medicine, Ewha Womans University College of Medicine, Seoul, South Korea; 8 Department of Rheumatology, Hanyang University Hospital for Rheumatic Diseases, Seoul, South Korea; 9 Division of Rheumatology, Department of Internal Medicine, Seoul St Mary’s Hospital, Center for Integrative Rheumatoid Transcriptomics and Dynamics, College of Medicine, the Catholic University of Korea, 222 Banpo-daero, Seocho-gu, Seoul, 06591 South Korea

**Keywords:** Rheumatoid arthritis, Tacrolimus, Tumor necrosis factor inhibitors, Maintenance, Low disease activity

## Abstract

**Background:**

Tapering or stopping biological disease-modifying anti-rheumatic drugs has been proposed for patients with rheumatoid arthritis (RA) in remission, but it frequently results in high rates of recurrence. This study evaluates the efficacy and safety of tacrolimus (TAC) as maintenance therapy in patients with established RA in remission after receiving combination therapy with tumor necrosis factor inhibitor (TNFi) and methotrexate (MTX).

**Methods:**

This 24-week, prospective, open-label trial included patients who received TNFi and MTX at stable doses for ≥24 weeks and had low disease activity (LDA), measured by Disease Activity Score-28 for ≥12 weeks. Patients selected one of two arms: maintenance (TNFi plus MTX) or switched (TAC plus MTX). The primary outcome was the difference in the proportion of patients maintaining LDA at week 24, which was assessed using a logistic regression model. Adverse events were monitored throughout the study period.

**Results:**

In efficacy analysis, 80 and 34 patients were included in the maintenance and switched arms, respectively. At week 24, LDA was maintained in 99% and 91% of patients in the maintenance and switched arms, respectively (odds ratio, 0.14; 95% confidence interval, 0.01–1.59). Drug-related adverse effects tended to be more common in the switched arm than in the maintenance arm (20.9% versus 7.1%, respectively) but were well-tolerated.

**Conclusion:**

This controlled study tested a novel treatment strategy of switching from TNFi to TAC in RA patients with sustained LDA, and the findings suggested that TNFi can be replaced with TAC in most patients without the patients experiencing flare-ups for at least 24 weeks.

**Trial registration:**

Korea CDC CRIS, KCT0005868. Registered 4 February 2021—retrospectively registered

**Supplementary Information:**

The online version contains supplementary material available at 10.1186/s13075-021-02566-z.

## Background

Rheumatoid arthritis (RA) is an autoimmune disease with progressive joint damage and deformities, eventually resulting in functional disability [1]. Due to early diagnosis, treat-to-target strategies, and effective disease-modifying anti-rheumatic drugs (DMARDs), remission is achievable, which can prevent or reduce the progression of joint damage and inflammation-related comorbidities [2–5]. Over the last three decades, targeted DMARDs have revolutionized RA therapeutics. The targets include several cytokines, specific lymphocyte subsets, cell-surface receptors, and signaling pathways. The first biological DMARDs (bDMARDs) inhibited tumor necrosis factor-α (TNFα) from binding to its receptors. TNFα is a central cytokine in the inflammatory cascade against infection and malignancies that promotes pannus formation and bone erosion in RA [6]. Since TNFα inhibitors (TNFi) were developed in the 1980s, five drugs with proven therapeutic efficacy and safety in RA have been used clinically [7–9].

Given the recent updates on RA management, we can consider tapering TNFi by dose reduction or prolonged intervals when the treatment is combined with conventional synthetic DMARDs (csDMARDs) [10]. The long-term use of TNFi is hindered by potential side effects (such as serious infections [11]), concerns of malignancies [12, 13], inconvenience of injections, and expense [14]. However, complete discontinuation of TNFi is not recommended because of the high recurrence rate (40–60%) [15–17].

It is unclear whether TNFi can be discontinued when RA flare-ups can be prevented by adding csDMARDs. Tacrolimus (TAC) is an immunosuppressant previously used to prevent rejection following organ transplantation and to treat autoimmune diseases, such as lupus nephritis and myasthenia gravis [18]. It is effective in RA and used as a csDMARD, mainly in the Asia-Pacific [19–21]. The efficacy of TAC against RA occurs via blockage of the calcineurin pathway in T-lymphocytes, inhibiting their proliferation and cytokine production [22]. An in vitro study demonstrated that TAC decreases the levels of inflammatory cytokines, including TNFα, in synoviocytes [23]. The therapeutic effects of TAC have been reported in the treatment of interstitial lung disease (ILD); hence, it is a treatment option against RA with ILD [24–26]. However, no prospective studies have investigated switching from bDMARDs to csDMARDs in patients with sustained RA remission. Studies that investigated de-escalating TNFi in patients with RA suggest that a constant degree of immunomodulation is not always required to maintain remission [27–31]. We conducted this prospective, non-randomized, active control, parallel group, open-label study to investigate the potential of stopping TNFi and adding TAC in patients with stable low disease activity (LDA).

## Patients and methods

### Study design

The “Anti-**T**NF agents versus tac**ro**limus as maintenance thera**p**y in r**h**eumatoid arthritis patients of inactive state receiving methotrexate concomitantl**y**” (TROPHY) study was a prospective, multicenter, non-randomized, active control, parallel group, 24-week trial. The study compared two therapeutic strategies, maintaining TNFi and switching to TAC following sustained LDA with TNFi (at least 6 months), at nine institutes in South Korea between November 18, 2012, and November 20, 2017. The primary objective was to evaluate the feasibility of switching from TNFi to TAC as maintenance therapy in RA patients with stable LDA following combination therapy with TNFi and methotrexate (MTX). The study protocol was approved by the Institutional Review Board of each participating institution. The study was conducted in accordance with the Korean Good Clinical Practice guidelines and the Helsinki Declaration. Written informed consent was obtained from all participants.

### Patients

Eligible patients were 20–70 years of age with established RA (≥ 12-month duration) according to 2010 American College of Rheumatology (ACR)/European League Against Rheumatism (EULAR) criteria [32]. The following were the inclusion criteria: (1) stable treatment with TNFi (etanercept, infliximab, and adalimumab) and MTX for ≥24 weeks without alterations in dose and interval for ≥12 weeks, (2) minimal MTX dose of 7.5 mg/week, (3) Disease Activity Score-28 (DAS28)-serum C-reactive protein (CRP) < 3.2 for ≥12 consecutive weeks before screening, (4) tender and swollen joints ≤5 based on the 66/68 Joint Count for four consecutive weeks before screening, and (5) erythrocyte sedimentation rate (ESR) < 28 mm/h or CRP < 1.0 mg/dL for four consecutive weeks before screening.

Patients who received DMARDs other than MTX and three TNFi within 4 weeks of screening were excluded. The use of oral glucocorticoids ≤10 mg/day was acceptable unless the dose was changed within 4 weeks before screening. Non-steroidal anti-inflammatory drugs were also allowed at the same dose if they were in use before baseline evaluation and the dosage was unchanged within 14 days prior to screening. Additionally, patients who had received intra-articular, intravenous, or intramuscular glucocorticoid injections or intra-articular hyaluronic acid injections within 4 weeks preceding screening were excluded. Patients with conditions such as cytopenia, transaminitis (> 2× upper normal limit), abnormal serum creatinine level (> 1.5× upper normal limit or > 2 mg/dL, whichever was smaller), hyperbilirubinemia (> 2× upper normal limit), and fasting glucose > 110 mg/dL or postprandial glucose > 200 mg/dL were excluded. Patients with a history of infection within 24 weeks prior to screening, patients with a history of malignancy (except cervical cancer or basal cell carcinoma that had completely responded to treatment more than 5 years prior to screening), pregnant or breastfeeding women, and women of childbearing age who were not using appropriate contraception were also excluded.

### Study patient number calculation

This trial was designed to detect equivalence of the proportion of patients who maintained LDA for 24 weeks between the two therapies. Based on historical data, 90% of patients on maintenance therapy maintain LDA after 24 weeks. For a priori sample-size estimation, it was assumed that the maintenance rate of switched therapy would be 70%. The expected difference between the two arms was fixed at 20%. The ratio of patients was set to 1:2 (switched vs. maintenance arm, respectively). With α = 5% and power = 80%, the required sample size was 48 and 96 patients in the switched and maintenance arms, respectively.

### Intervention

**Fig. 1:**
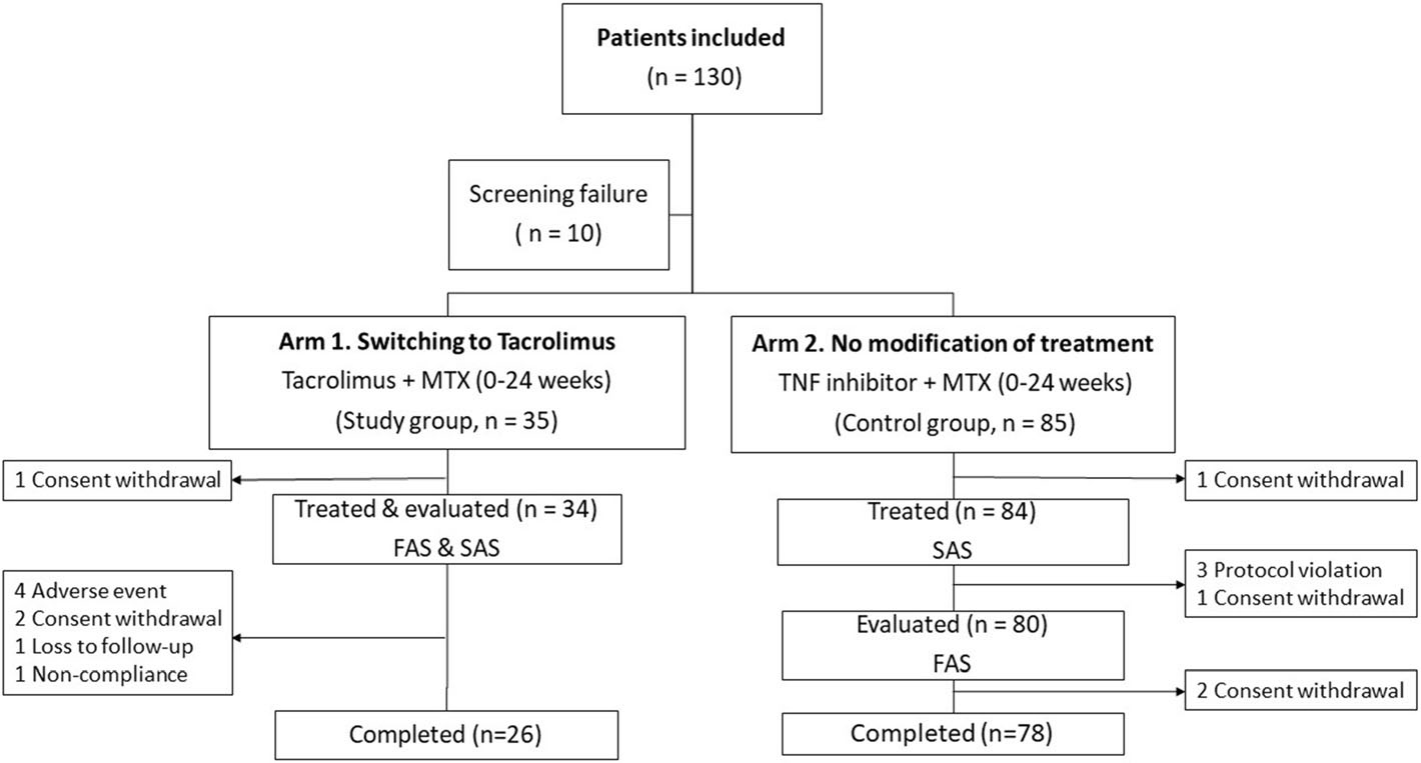
Flow chart of the patients enrolled in the TROPHY study. Overall, 130 patients with rheumatoid arthritis were screened and 120 patients with sustained low disease activity (Disease Activity Score-28 ≤ 3.2) with tumor necrosis factor inhibitor (TNFi) plus methotrexate (MTX) were divided into two treatment arms that either switched from TNFi to tacrolimus (TAC) or maintained the same treatment. Efficacy was evaluated in the full analysis set (FAS) and safety was evaluated in all patients who received at least one dose of TAC or TNFi (safety analysis set, SAS)

Patients chose to either change from TNFi to TAC (TAC + MTX; switched arm) or maintain TNFi (TNFi+MTX; maintenance arm) after understanding the differences between the treatments. In the switched arm, TNFi was switched to TAC (1 mg/day orally), which was increased to 3 mg/day at the investigator’s discretion. The maintenance arm continued to receive TNFi at the standard dose (etanercept, 50 mg weekly; adalimumab, 40 mg every other week; infliximab, 5 mg/kg every 8 weeks) or reduced dose for 24 weeks. In both arms, MTX was maintained at a constant dose throughout the study but dose reduction was permitted for adverse events (AEs). The patients were evaluated at baseline, 8, 16, and 24 weeks. The switched arm included additional visits at 2 and 4 weeks (Fig. 1).

### Efficacy measurements

The primary endpoint was the proportion of patients who maintained LDA at week 24. Disease activity was assessed using DAS28-CRP every 8 weeks at each center by a rheumatologist who was blinded to the patient group. The secondary endpoints included the proportion of patients who maintained LDA at weeks 8 and 16; the remission rate at weeks 8, 16, and 24; and the Health Assessment Questionnaire Disability Index (HAQ-DI) at week 24. Functional ability was measured using HAQ-DI every 8 weeks. Additionally, the progression of structural damage was assessed using the method of Larsen et al. [33], which relies on plain radiographs of the hands and feet that were assessed by two independent readers at each site.

Exploratory outcomes included tender joint count in 68 joints (TJC68), swollen joint count in 66 joints (SJC66), serum ESR and CRP, Physician’s Global Assessment of Disease Activity (PhGA), and Patient Global Assessment of Disease Activity (PGA) based on a visual analog scale (VAS) of 0–100.

### Safety profile

Safety data were obtained through interviews, physical examinations, and laboratory tests at each visit. Safety variables included all treatment-emergent adverse events (TEAEs) that occurred after administration of TAC or TNFi, serious AEs, AEs of special interest (infections, malignancy, and gastrointestinal disorders), and laboratory parameters. The association of AEs and laboratory abnormalities with the drugs was entirely based on the clinical judgment of the investigator.

### Statistical analysis

Demographic and efficacy analyses included patients who received at least one dose of TAC or TNFi and were evaluated for efficacy. Safety analyses included all patients who received at least one dose of TAC or TNFi irrespective of efficacy evaluation (Fig. 1). Baseline characteristics were analyzed using chi-square tests or Fisher’s exact tests for categorical variables and two-sample t-tests or Wilcoxon rank-sum tests for continuous variables, as appropriate. Descriptive results are presented as means ± standard deviations.

For the primary and secondary endpoints, the difference in the proportion of patients in remission or LDA between the groups was compared using a logistic regression model with adjustments for baseline DAS28, with the results expressed as odds ratios (ORs) and 95% confidential intervals (CIs). Paired t-tests or Wilcoxon signed-rank tests were used to compare DAS28, HAQ-DI, and other ACR Core Data Sets from baseline scores within each group. The differences in these scores between the treatment arms at each visit were assessed using analysis of covariance (ANCOVA) with the baseline value of each parameter as a covariate. Results are summarized as least square (LS) mean differences and 95% CIs. A Kaplan–Meier plot was used to illustrate flare-ups (DAS28 > 3.2) over 24 weeks according to the allocation of the study arm. All analyses were performed using SAS 7.4 (SAS Institute, Cary, NC, USA) and *P*-values < 0.05 were considered statistically significant.

## Results

### Demographic and baseline characteristics

Enrolment was lower than expected; 130 patients were screened, and 120 patients were enrolled in the switched arm (n = 35) and maintenance arm (n = 85) (Fig. 1). The patients were 22–70 years of age and included 95 (83.3%) women. The duration of RA was 13–301 months, and all patients were seropositive. The baseline demographic data were not significantly different between the two arms (Table 1). Overall, 118 (98.3%) patients received medications. In the maintenance arm (n = 84), three patients withdrew consent and three were excluded for protocol violations (exclusion criteria not met); therefore, 78 patients were included. In the switched arm, eight patients dropped out, four developed AEs, two withdrew consent, one had a protocol deviation, and one was lost to follow-up. The proportion of patients who completed the study was lower in the switched arm (n = 26, 74.2%) than in the maintenance arm (n = 78, 91.8%).

**Table 1:** Baseline characteristics

Parameters	TAC + MTX (n = 34)	TNFi + MTX (n = 80)	***P-***value
Age, years	51.3 ± 9.7	50.5 ± 10.9	0.8598
Females, n (%)	31 (91.2)	64 (80.0)	0.1430
Body mass index, kg/m^2^	23.0 ± 2.5	23.0 ± 2.8	0.9284
Disease duration, months	95.0 ± 50.8	90.3 ± 70.0	0.2797
RF positivity, n (%)	27 (79.4)	65 (81.2)	0.8219
ACPA positivity, n (%)	32 (94.1)	64 (80)	0.0594
Tender joint count (0–68)	0.2 ± 0.7	0.2 ± 0.4	0.2760
Swollen joint count (0–66)	0.1 ± 0.5	0.1 ± 0.3	0.8964
PhGA (VAS, mm)	9.3 ± 10.6	10.0 ± 8.3	0.7022
PGA (VAS, mm)	15.4 ± 14.3	17.3 ± 17.9	0.5827
ESR, mm/h	21.3 ± 14.9	20.6 ± 13.5	0.8092
CRP, mg/dL	0.31 ± 0.48	0.23 ± 0.30	0.2833
DAS28-CRP	1.28 ± 0.48	1.25 ± 0.29	0.6820
HAQ-DI	0.28 ± 0.32	0.43 ± 0.50	0.1097
Larsen score	12.5 ± 19.1	12.1 ± 20.5	0.9190
MTX dose, mg/week	10.0 ± 2.3	10.8 ± 2.9	0.1398
Glucocorticoids, n (%)	16 (47.1)	43 (53.8)	0.5173

Continuous variables are presented as means ± standard deviations unless otherwise indicated. *ACPA*, anti-citrullinated peptide antibody; *CRP*, C-reactive protein; *DAS28-CRP*, Disease Activity Score for 28 joints based on CRP; *ESR*, erythrocyte sedimentation rate; *HAQ-DI*, Health Assessment Questionnaire Disability Index; *MTX*, methotrexate; *PhGA*, Physician’s Global Assessment of Disease Activity; *PGA*, Patient’s Global Assessment of Disease Activity; *RF*, rheumatoid factor; *TNFi*, tumor necrosis factor inhibitor; *VAS*, visual analog scale; *TAC*, tacrolimus

### Primary and major secondary efficacy

**Fig. 2:**
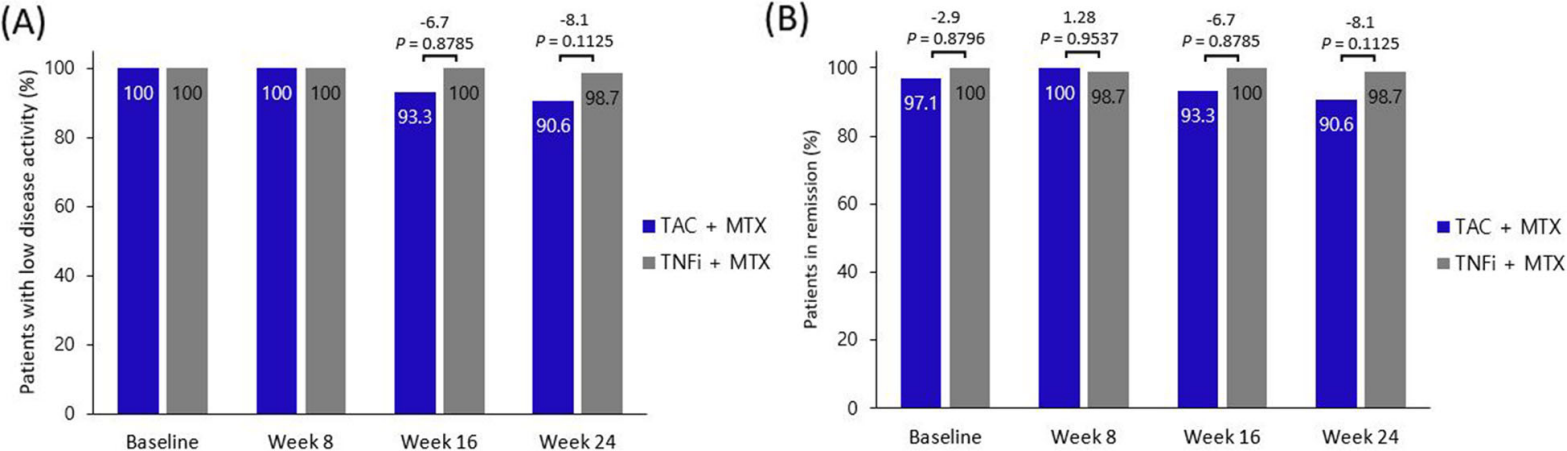
Comparison of the proportion of patients with sustained low disease activity (**A**) and remission (**B**) at baseline and weeks 8, 16, and 24. Bars indicate the percentage of patients, while data in the bars are the proportion of patients. The difference in proportion between the two arms and *P*-value of the logistic regression after adjusting for baseline Disease Activity Score-28 are shown above the bars. TAC, tacrolimus; TNFi, tumor necrosis factor inhibitor; MTX, methotrexate

Overall, 114 (97%) patients were evaluated for efficacy (Fig. 1). At week 24, a comparable number of patients in the switched and maintenance arms maintained LDA (DAS28 < 3.2) (29/32 [90.6%] vs. 79/80 [98.7%], respectively) (Fig. 2A). After adjustment for baseline DAS28, the proportion of patients who maintained LDA or remission in the two arms was not significantly different (OR, 0.142; 95% CI, 0.013–1.566; *P* = 0.111). The mean change in DAS28 between baseline and week 24 in the switched and maintenance arms was 0.30 ± 0.87 and 0.10 ± 0.50, respectively. The LS mean difference between the arms was 0.21 (95% CI, −0.04–0.47, *P* = 0.103).

The maintenance rates of LDA at weeks 8 and 16 were 100% and 93.3% in the switched arm and 100% and 100% in the maintenance arm, respectively. The remission (DAS28 ≤ 2.6) rate was not different between the arms at weeks 8, 16, and 24 (switched arm, 100%, 93.3%, and 90.6%, respectively; maintenance arm, 98.7%, 100%, and 98.7%, respectively) (Fig. 2B). In this study, the TNFi dose was de-escalated in 11.3% of patients in the maintenance arm, and none of them developed a relapse.

**Fig. 3:**
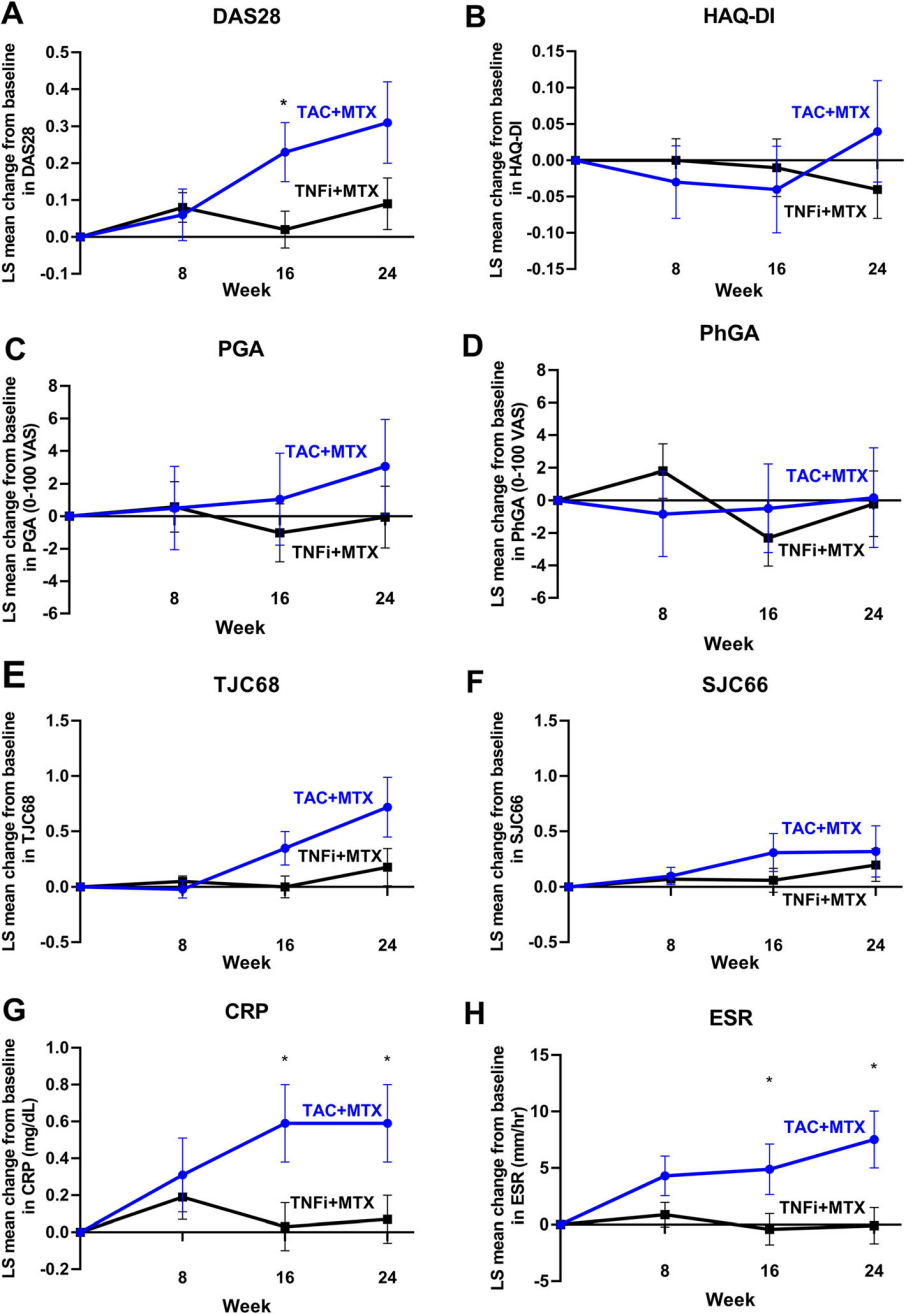
Evolution of disease activity indices during each week over 24 weeks of follow-up compared with the baseline for **A** DAS28-CRP, **B** HAQ-DI, **C** PGA, **D** PhGA, **E** TJC68, **F** SJC66, **G** CRP, and **H** ESR. ^*^P < 0.05. CRP, C-reactive protein; DAS28, Disease Activity Score in 28 joints; ESR, erythrocyte sedimentation rate; HAQ-DI, Health Assessment Questionnaire Disability Index; MTX, methotrexate; PGA, Patient’s Global Assessment of Disease Activity; PhGA, Physician’s Global Assessment of Disease Activity; SGAP, Subject’s Global Assessment of Pain; SJC66, swollen joint count at 66 joints; TAC, tacrolimus; TJC68, tender joint count at 68 joints; TNFi, tumor necrosis factor inhibitor; VAS, visual analog scale; LS, least squares

The mean difference in change in DAS28 between the arms after adjusting for baseline DAS28 was significant at week 16 (0.22; 95% CI, 0.02–0.41, *P* = 0.0285) (Fig. 3A). HAQ-DI, PGA, PhGA, TJC68, and SJC66 did not change significantly from baseline in both arms at weeks 8, 16, and 24 (Fig. 3B–F). However, the mean change in serum CRP between baseline and weeks 16 and 24 was higher in the switched arm than in the maintenance arm (week 16, 0.58 ± 2.18 vs. 0.04 ± 0.31, respectively, *P* = 0.0283; week 24, 0.58 ± 2.10 vs. 0.08 ± 0.44, respectively, *P* = 0.0398) (Fig. 3G). The change in serum ESR also was higher in the switched arm than in the maintenance arm at week 16 (4.53 ± 14.63 vs. −0.28 ± 12.59, respectively, *P* = 0.0460) and week 24 (7.31 ± 19.16 vs. 0 ± 12.66, respectively, *P* = 0.0121) (Fig. 3H).

**Fig. 4:**
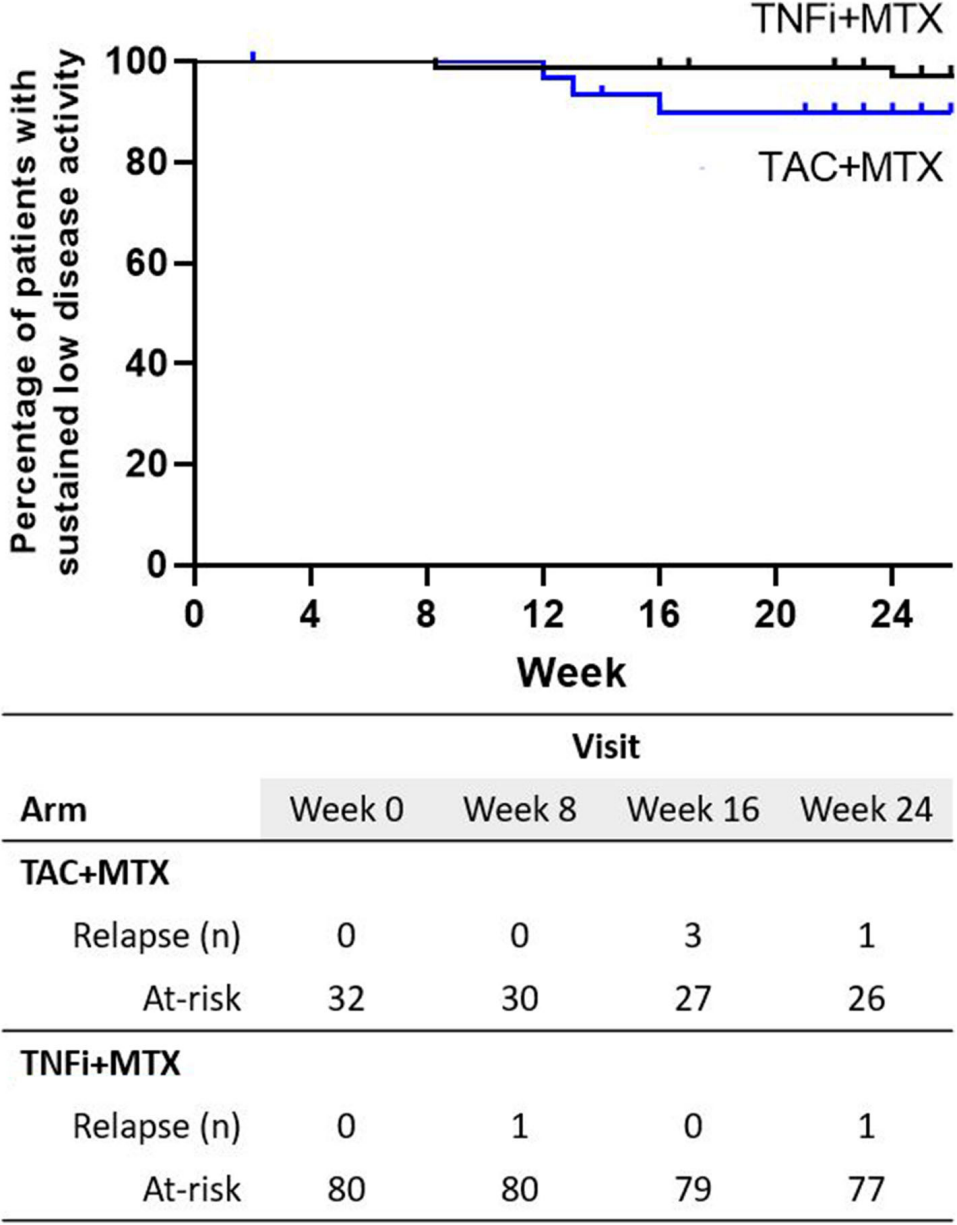
Kaplan–Meier curve for maintenance of low disease activity. Curves indicate loss of low disease activity over 24 weeks in relation to the treatment arms (blue: switching to tacrolimus; black: continuing TNFi). MTX, methotrexate; TAC, tacrolimus; TNFi, tumor necrosis factor inhibitor

Five patients developed flare-ups (DAS28 > 3.2) over 24 weeks, three in the switched arm and two in the maintenance arm (*P* = 0.1398). The cumulative incidence curves of flare-ups are illustrated in Fig. 4. The risk of flare-ups was not different between the two arms (hazard ratio [HR], 3.628; 95% CI, 0.579–22.714; *P* = 0.1685). The unadjusted mean time to flare-up was 16 and 24 weeks in the switched and maintenance arms, respectively. No radiographic changes were noted in both arms for 6 months (Supplemental Fig. 1 in Additional file 1).

### Adverse events

During the study, 42 cases of TEAEs occurred in 35 (29.7%) patients including 16/34 patients (47.1%, 17 cases) in the switched arm and 19/84 patients (22.6%, 25 cases) in the maintenance arm (*P* = 0.0085). Drug-related AEs were reported in 7/34 patients (20.6%, 7 cases) in the switched arm and 6/84 patients (7.1%, 7 cases) in the maintenance arm (*P* = 0.0501) (Table 2). The most common TAC-associated AE was abdominal pain (11.7%, 4 cases). Infection was reported in one patient (2.9%) in the switched arm and four patients (4.8%) in the maintenance arm (*P* > 0.999). One patient in the switched arm was diagnosed with disseminated tuberculosis, which was the only severe adverse drug reaction in this study, and four patients in the maintenance group reported upper respiratory and oral herpes simplex infections. Major hematological and biochemical abnormalities were not observed in either arm. No death or malignancy was reported in either arm.

**Table 2:** Summary of drug-related treatment-emergent adverse events

	Tacrolimus + MTX (n = 34)	TNFi + MTX (n = 84)	***P***-value
**TEAE, n (%)**	16 (47.1)	19 (22.6)	0.0085
**Adverse drug reaction, n (%)**	7 (20.6)	6 (7.1)	0.0501
*Infections and infestations*	1 (2.9)	4 (4.8)	
Upper respiratory tract infection	0	3 (2.5)	
Disseminated tuberculosis	1 (2.9)	0	
Oral herpes simplex infection	0	1 (1.19)	
*Gastrointestinal disorders*	4 (11.76)	0	
Abdominal pain	4 (11.76)	0	
*Musculoskeletal disorders*	1 (2.9)	1 (1.2)	
*Lymphatic system disorders*	0	1 (1.19)	
Lymphadenopathy	0	1 (1.19)	
*Nervous system disorders*	1 (2.94)	0	
Headache	1 (2.94)	0	

Each value is presented as number (%). *MTX*, methotrexate; *TNFi*, tumor necrosis factor inhibitor; *TEAE*, treatment-emergent adverse event

## Discussion

Tapering or stopping DMARDs is important for patients and rheumatologists since longer remission is achieved in more patients due to better treatments. Once remission is achieved, DMARD down-titration is considered based on patient preferences, safety issues, and/or economic reasons [13–15, 34]. Previous studies have indicated that LDA was maintained in only 40–60% and 50–80% of patients with TNFi discontinuation [17, 29, 35–37] and de-escalation, respectively [29, 35, 36, 38]. Several factors are considered as predictors of disease relapse after tapering or stopping TNFi. One of them is remission quality; Tanaka et al. reported that the relapse rate in patients who maintained “deep remission” (DAS28-ESR ≤ 1.98) was relatively low (21%) [37]. Another factor is the presence of anti-citrullinated protein antibodies (ACPAs); relapses observed within 6 months of DMARD de-escalation were associated with ACPAs [29].

Although more than half the patients were able to maintain LDA despite de-escalation of treatment, a considerable number of them developed relapse during tapering of TNFi [28, 29, 35, 36]. De-escalation of TNFi therapy cannot be free from considerations of safety and costs. For example, active tuberculosis has been reported in patients who received preventive treatment for latent tuberculosis before starting TNFi as well as in those with negative test results for latent tuberculosis infection [39]. The present study is, to our knowledge, the first trial to implement the concept of using csDMARDs as substitutes for bDMARDs in patients with LDA on combined bDMARD and MTX therapy. LDA was observed in similar proportions of patients in both arms at week 24. Our findings provide new clinical evidence that TNFi can be switched to other oral csDMARDs, such as TAC due to the safety and cost concerns of TNFi, in patients who achieve LDA, particularly those at risk of tuberculosis recurrence.

TAC inhibits T-cell activation and has been used as a second-line DMARD for RA [21]. Our group has demonstrated that it also markedly suppresses TNF production by rheumatoid synoviocytes [23]. In the present study, TNFi was replaced with TAC because (i) patients in this study were treated with ≥2 csDMARDs (including MTX) for ≥6 months before TNFi and failed to respond and (ii) TNFi and TAC possess some mechanistic overlap in their drug actions [40]. Moreover, TAC demonstrated non-inferior efficacy and tolerability compared with leflunomide [20]. Additionally, TAC is administered orally, which is convenient. Financially, 1.5 mg TAC costs approximately 1675 USD/year, whereas the standard dose of TNFi costs 4489–8949 USD/year in South Korea (cost details in other countries are listed in Supplemental Table 1 in Additional file 1). Therefore, TAC can be preferred when tapering or stopping TNFi.

Due to enrolment difficulties, especially for the switching arm, 5 years was needed to recruit sufficient numbers of patients for this study. The main reason for the enrolment difficulties was that patients in remission with TNFi were less willing to discontinue TNFi and switch to another drug that they had never used. Notwithstanding the tremendous difficulty in patient recruitment, we demonstrated that the proportion of patients with LDA over 24 weeks who switched to TAC was not different from that of patients who continued TNFi. Specifically, 91% of patients who switched to TAC and 99% of those who continued TNFi maintained LDA over 24 weeks.

While several efficacy variables did not increase significantly in the switched arm, CRP and ESR were significantly elevated in this arm compared with the maintenance arm at weeks 16 and 24. Berkhout et al. [41] reported that circulating TNF levels increased during TNFi treatment, but were inactivated by binding with TNFi. They also found that TNFi discontinuation resulted in a rapid decrease in the concentration of TNFi (90% reduction) at week 12, while the TNFα concentration decreased by only 25%. Therefore, one possible explanation for our finding is that the sudden discontinuation of TNFi resulted in rapid increases in TNF levels and inflammation. Moreover, TNFα affects pain responses in the central nervous system [42], and discontinuation of TNFi could be associated with temporary increases in pain perception and disease activity. Finally, TAC (2–3 mg) is effective as a monotherapy in patients with RA in a dose-dependent manner [43], and the average dose of TAC in our study was 1.45 mg/day; therefore, steady-state concentrations of TAC may not have been reached in some patients. Accordingly, tapering the TNFi for 2–3 months and overlapping this with TAC titration may be needed to reduce temporary RA flare-ups after discontinuation of TNFi.

Regarding safety, gastrointestinal symptoms, such as abdominal pain, were the most common issue in the switched arm, which is similar to symptoms previously reported, including diarrhea, nausea, abdominal pain, and dyspepsia [44]. Decreased kidney function is an important concern with TAC therapy; however, elevated serum creatinine was not observed in our study over 24 weeks.

This study has some limitations. First, the sample size was small; the patient pool in the switched arm was less than planned. Enrolment difficulties might have been caused by the unwillingness of patients to change effective medications that they had used for > 6 months. With a larger sample size, further differences in the efficacy and safety between the switched and maintenance arms could have been observed. Second, non-randomization and lack of blinding, which were not possible due to ethical reasons, could have influenced the outcomes. Participant bias might have influenced our results, especially when certain patients wished to maintain TNFi treatment or preferred oral drug administration route rather than injections. Third, although flare-ups have been reported 15 weeks after TNFi discontinuation [45], 24 weeks is a short period to assess potential radiographic progression. Further long-term studies are needed to identify patients who could benefit from this treatment strategy and establish better switching and discontinuation strategies.

This trial has several strengths. To the best of our knowledge, this is the first study to suggest a strategy of switching from bDMARDs to TAC following stable remission with bDMARDs, including TNFi. It included patients in whom csDMARDs had failed at least 6 months before TNFi and who had longer disease duration and positive ACPAs, which collectively suggests progressive RA. Nevertheless, the flare-up rate in the group that switched from TNFi to TAC was much lower (9.0%) than that in previously reported studies on tapering TNFi without rescue medication.

## Conclusions

The TROPHY study provides a new perspective on managing RA patients with stable LDA or those in remission. Switching to TAC and discontinuing TNFi is feasible, and most patients maintained LDA over 24 weeks.

## Supplementary Information

**Additional file 1:.** Supplemental Figure 1 and Table 1

**Additional file 2:.** Supplementary methods and results

## Data Availability

All data generated or analyzed during this study are included in this published article and supplemental materials.

## Abbreviations

ACR: American College of Rheumatology
AE: Adverse event
bDMARD: Biological disease-modifying anti-rheumatic drug
CI: Confidence interval
CRP: C-reactive protein
csDMARD: Conventional synthetic disease-modifying anti-rheumatic drug
DAS28: Disease Activity Score in 28 joints
EULAR: European League Against Rheumatism
ESR: Erythrocyte sedimentation rate
LDA: Low disease activity
PhGA: Physician’s Global Assessment of Disease Activity
PGA: Patient Global Assessment of Disease Activity
RA: Rheumatoid arthritis
SJC66: Swollen joint count in 66 joints
TAC: Tacrolimus
TEAE: Treatment-emergent adverse event
TNFi: Tumor necrosis factor inhibitor
TJC68: Tender joint count in 68 joints
VAS: Visual analog scale
