# Supplementary material for: Switching from TNFα inhibitor to tacrolimus as maintenance therapy in rheumatoid arthritis after achieving low disease activity with TNFα inhibitors and methotrexate: 24-week result from a non-randomized, prospective, active-controlled trial
Source: Arthritis Res Ther. 2021 Jul 8;23:182. doi: 10.1186/s13075-021-02566-z (PMC8265052; doi:10.1186/s13075-021-02566-z)
Supplement: Supplementary file 1 — Additional file 1:. Supplemental Figure 1 and Table 1 [file 13075_2021_2566_MOESM1_ESM.docx]

**Additional File 1.**

**Switching from TNFα inhibitor to tacrolimus as maintenance therapy** **in rheumatoid arthritis after achieving low disease activity with TNFα inhibitors and methotrexate: 24-week result from a non-randomized, prospective, active-controlled trial**

Sang Youn Jung^1,a^, Jung Hee Koh^2,a^, Ki-Jo Kim^3^, Yong-Wook Park^4^, Hyung-In Yang^5^,

Sung Jae Choi^6^, Jisoo Lee^7^, Chan-Bum Choi^8^, Wan-Uk Kim^9*^

^1^ Division of Rheumatology, Department of Internal Medicine, CHA Bundang Medical Center, CHA University, Seongnam, Korea; ^2^ Division of Rheumatology, Department of Internal Medicine, Bucheon St. Mary's Hospital, the Catholic University of Korea, Seoul, Korea; ^3^ Division of Rheumatology, Department of Internal Medicine, St. Vincent Hospital, the Catholic University of Korea, Seoul, Korea; ^4^ Division of Rheumatology, Department of Internal Medicine, Chonnam National University Medical School and Hospital, Gwangju, Korea; ^5^ Division of Rheumatology, Department of Internal Medicine, Kyung Hee University College of Medicine, Kyung Hee University Hospital at Gangdong, Seoul, Korea; ^6^ Division of Rheumatology, Department of Internal Medicine, Korea University Ansan Hospital, Ansan, Korea; ^7^ Division of Rheumatology, Department of Internal Medicine, Ewha Womans University College of Medicine, Seoul, Korea; ^8^ Department of Rheumatology, Hanyang University Hospital for Rheumatic Diseases, Seoul, Korea; ^9^ Division of Rheumatology, Department of Internal Medicine, Seoul St Mary's Hospital, the Catholic University of Korea, Seoul, Korea

^a^ These authors contributed equally to this work.

***Correspondence to:**

Professor Wan-Uk Kim, M.D., Ph.D.

Division of Rheumatology, Department of Internal Medicine, Seoul St. Mary's Hospital

Center for Integrative Rheumatoid Transcriptomics and Dynamics, College of Medicine

The Catholic University of Korea, 222 Banpo-daero, Seocho-gu, Seoul 06591

Tel.: 82-2-2258-7530

Fax: 82-2-2258-7526

Email: [wan725@catholic.ac.kr](mailto:wan725@catholic.ac.kr)

**Supplemental Figure 1.** Evolution of radiographic damage at 24 weeks of follow-up compared with baseline as determine using the Larsen score.

LS, lean square mean; MTX, methotrexate; TAC, tacrolimus; TNFi, Tumor necrosis factor inhibitor

**Supplemental Table 1.** The average annual cost of tacrolimus and TNFi

|  |  | Korea | Japan | US | Germany |
| --- | --- | --- | --- | --- | --- |
| Tacrolimus | 1 mg | 1,117 | 1,894 | 2,833 | 2,225 |
|  | 2 mg | 2,233 | 3,787 | 5,666 | 4,450 |
|  | 3 mg | 3,350 | 5,681 | 8,498 | 6,675 |
| Etanercept | 50 mg/syringe | 6,402 | 11,676 | 72,240 | 15,114 |
| Adalimumab | 40 mg/syringe | 8,949 | 14,506 | 93,104 | 15,698 |
| Remicade | 100 mg/vial  60 kg adult | 4,489  8,978 | 4,222  8.222 | 6,419  12,838 | 5,536  11,072 |

The base currency is USD, the reference time is May 2021, which was the exchange rate period.
