# Supplementary material for: Switching from TNFα inhibitor to tacrolimus as maintenance therapy in rheumatoid arthritis after achieving low disease activity with TNFα inhibitors and methotrexate: 24-week result from a non-randomized, prospective, active-controlled trial
Source: Arthritis Res Ther. 2021 Jul 8;23:182. doi: 10.1186/s13075-021-02566-z (PMC8265052; doi:10.1186/s13075-021-02566-z)
Supplement: Supplementary file 2 — Additional file 2:. Supplementary methods and results [file 13075_2021_2566_MOESM2_ESM.docx]

**Additional File 2.**

**Switching from TNFα inhibitor to tacrolimus as maintenance therapy** **in rheumatoid arthritis after achieving low disease activity with TNFα inhibitors and methotrexate: 24-week result from a non-randomized active-controlled trial**

Sang Youn Jung^1,a^, Jung Hee Koh^2,a^, Ki-Jo Kim^3^, Yong-Wook Park^4^, Hyung-In Yang^5^,

Sung Jae Choi^6^, Jisoo Lee^7^, Chan-Bum Choi^8^, Wan-Uk Kim^9*^

^1^ Division of Rheumatology, Department of Internal Medicine, CHA Bundang Medical Center, CHA University, Seongnam, Korea; ^2^ Division of Rheumatology, Department of Internal Medicine, Bucheon St. Mary's Hospital, the Catholic University of Korea, Seoul, Korea; ^3^ Division of Rheumatology, Department of Internal Medicine, St. Vincent Hospital, the Catholic University of Korea, Seoul, Korea; ^4^ Division of Rheumatology, Department of Internal Medicine, Chonnam National University Medical School and Hospital, Gwangju, Korea; ^5^ Division of Rheumatology, Department of Internal Medicine, Kyung Hee University College of Medicine, Kyung Hee University Hospital at Gangdong, Seoul, Korea; ^6^ Division of Rheumatology, Department of Internal Medicine, Korea University Ansan Hospital, Ansan, Korea; ^7^ Division of Rheumatology, Department of Internal Medicine, Ewha Womans University College of Medicine, Seoul, Korea; ^8^ Department of Rheumatology, Hanyang University Hospital for Rheumatic Diseases, Seoul, Korea; ^9^ Division of Rheumatology, Department of Internal Medicine, Seoul St Mary's Hospital, the Catholic University of Korea, Seoul, Korea

^a^ These authors contributed equally to this work.

***Correspondence to:**

Professor Wan-Uk Kim, M.D., Ph.D.

Division of Rheumatology, Department of Internal Medicine, Seoul St. Mary's Hospital

Center for Integrative Rheumatoid Transcriptomics and Dynamics, College of Medicine

The Catholic University of Korea, 222 Banpo-daero, Seocho-gu, Seoul 06591

Tel.: 82-2-2258-7530

Fax: 82-2-2258-7526

Email: [wan725@catholic.ac.kr](mailto:wan725@catholic.ac.kr)

**Contents**

**Supplementary Methods3**

Summary of study protocol3

Schedule of assessments 13

**Supplementary Results15**

Demographic Data15

Table 1.1 Subject disposition (Screened Set)15

Table 1.2 Analysis Sets (Enrolled Set)16

Table 1.3 Major Protocol Deviation (Enrolled Set)17

Table 1.4 Medical History by SOC and PT (Full Analysis Set)18

Table 1.5 Treatment compliance (Full Analysis Set)19

Efficacy Data20

Table 2.1 Summary of Morning Stiffness (Per-Protocol Set)20

Table 2.2 Summary of Larsen Score (Per-Protocol Set)24

Safety Data26

Table 3.1 Extent of Exposure (Safety Set)26

Table 3.2 Overall Summary of TEAE (Safety Set)27

Table 3.3 Overall Summary of ADR (Safety Set)29

Table 3.4 Incidence of TEAE by SOC and PT (Safety Set)31

Table 3.5 Incidence of ADR by SOC and PT (Safety Set)34

Table 3.6 Incidence of TEAE by Maximum Severity, SOC and PT (Safety Set)35

Table 3.7 Incidence of ADR by Maximum Severity, SOC and PT (Safety Set)38

Table 3.8 Incidence of TEAE by Maximum Relationship, SOC and PT (Safety Set)39

**Summary of Protocol**

| **Title** | A multicenter, non-randomized, active controlled, parallel group, open, 24-week study to assess the efficacy and safety of TNF inhibitors versus tacrolimus as maintenance therapy in rheumatoid arthritis patients of inactive state receiving methotrexate concomitantly (Trophy study) |
| --- | --- |
| **Stage and design** | A multicenter, non-randomized, active controlled, parallel group, open, 24-week study, Investigator-initiated trial  Sample size estimation  This trial was designed to detect equivalence of the proportion of patients who maintained LDA for 24 weeks between the two therapies. Based on historical data, 90% of patients on maintenance therapy maintain LDA after 24 weeks. For a priori sample-size estimation, it was assumed that the maintenance rate of switched therapy would be 70%. The non-inferiority margin for the difference was fixed at 20%. The ratio of patients was set to 1:2 (switched vs. maintenance arm, respectively). With α=5% and power=80%, the required sample size was 48 and 96 patients in the switched (Tacrolimus) and maintenance arms (TNFi), respectively. |
| **Objectives** | Background  In patients with RA who achieve sustained remission with biological DMARDs, stopping or tapering biological DMARDs can be considered when the treatment is combined with a conventional synthetic DMARD. Previous clinical trials that have investigated cessation of TNFi have reported high rates of RA recurrence.  1) Primary objective   - To evaluate the feasibility of switching from TNFi to tacrolimus as maintenance therapy in patients with RA with stable LDA following combination therapy with TNFi and MTX   2) Secondary objectives   - 1. Safety of switching from TNFi to tacrolimus   2. Assessment of physical function and disability   3. Measuring health outcomes |
| **Subjects** | Patients diagnosed with RA who fulfilled the classification criteria of ACR/EULAR in 2010 |
| **Inclusion and exclusion criteria** | **Inclusion criteria**  1) Patients with RA for at least 12 months to less than 15 years  2) 20–70 years of age at the time of the screening visit  3) Patients who received TNFi for ≥24 weeks and had DAS28-CRP < 3.2 for ≥ 12 consecutive weeks before screening  4) Subjects who can understand the information provided to them and who can voluntarily sign written consent  5) Subjects who understand the difference between tacrolimus treatment and TNFi treatment and can choose their treatment  6) If a female of child-bearing age, a negative pregnancy test at the time of the screening visit   1. For female subjects, there must be at least one year of menopause, surgical sterilization, or effective use of acceptable contraception methods. For contraception, subjects must agree to use an appropriate method during the clinical trial period and for 12 weeks after the end of administration of the investigational drug. 2. For male subjects, the subject or subject's female partner must agree to use an appropriate method of contraception during the trial period and for 12 weeks after the end of the investigational drug administration.   7) Subjects with LDA (as defined below) at screening and baseline:  A. Stable treatment with TNFi and MTX for ≥24 weeks without alterations in dose and interval for ≥12 weeks  B. ESR <28 mm/h or CRP <10 mg/dL for at least 4 consecutive weeks before screening  C. Tender and swollen joint count ≤ 5 for at least 4 consecutive weeks before screening  8) Subjects who received TNFi and MTX treatment (regardless of folic acid administration) for at least 24 weeks prior to baseline visit. The minimum acceptable stabilizing dose of MTX is 7.5 mg per week.  **Exclusion criteria**  **Exclusion criteria related to RA**  1) Subjects diagnosed with other forms of inflammatory arthritis (e.g., psoriatic arthritis, ankylosing spondylitis, or reactive arthritis)  2) Subjects with secondary noninflammatory arthritis (e.g., osteoarthritis or fibromyalgia) with symptoms sufficient to interfere with evaluation of the efficacy of the investigational drug for the main diagnosed RA disease, in the opinion of the investigator  3) Subjects who continue to have prostheses that have been infected at least once  **Exclusion criteria for concomitant drugs**  Subjects who have used the following medications for a certain period before the baseline visit   \| Classification of drug \| \| Dosage \| Exclusion \| \| --- \| --- \| --- \| --- \| \| Concomitant medication \| Analgesics \| Any dose \| Used within 24 h prior to baseline arthritis assessment \| \| NSAIDs  /COX-2 Inhibitors \| Any dose \| Change in dosage within 14 days prior to baseline arthritis assessment \| \| Oral corticosteroids \| Allowed if maximum daily dose ≤10 mg (or equivalent) of prednisolone \| Change in dosage within 28 days prior to baseline arthritis assessment \| \| Contraindicated medications \| IM/IV/IA  corticosteroids \| Any dose \| Used within 28 days prior to baseline arthritis assessment \| \| IA hyaluronic acid \| Any dose \| Used within 28 days prior to baseline arthritis assessment \| \| (DMARDs)  sulfasalazine  azathioprine  cyclosporin  cyclophosphamide  hydroxychloroquine  mizoribine  bucillamine  leflunomide  mycophenolate mofetil  tacrolimus \| Any dose \| Used within 28 days prior to baseline arthritis assessment \|   **Exclusion criteria related to medical history**  1) Female subjects who are lactating, pregnant, or planning to become pregnant during the clinical trial period or for 12 weeks after the administration of the investigational drug  2) Subjects with a history of chronic infection, recent severe infection, or life-threatening infection (within 24 weeks, including shingles), or signs or symptoms that may be considered infection (e.g., fever, cough)  3) In the opinion of the investigator, subjects with high risk of infection  4) Subjects who received any live vaccine within 8 weeks prior to baseline visit  5) Subjects known to be infected with HIV  6) Subjects with a history of lymphoma or lymphoproliferative disease, or subjects with signs and symptoms suggestive of lymphoproliferative disease  7) Subjects with active malignancies or a history of malignant tumors. However, cervical cancer or basal cell carcinoma that has completely responded to treatment for >5 years before screening is allowed.  8) Subjects with a current or recent history of severe, ongoing, and/or uncontrolled kidney, liver, blood, gastrointestinal, endocrine, lung, heart, neurological, or brain disease and deemed inappropriate for clinical trial selection by the investigator.  9) Subjects with NYHA grade III or IV congestive heart failure  10) Subjects diagnosed with systemic inflammatory diseases (e.g., scleroderma, systemic lupus erythematosus, and mixed connective tissue disease)  11) Subjects with positive hepatitis B surface antigen test and/or hepatitis C antibody test results  12) Subjects with current or history of central nervous system disease, demyelinating disease, or convulsive disease (e.g., multiple sclerosis, and epilepsy)  13) Subjects who are scheduled for or need a surgical joint procedure  14) Persons considered to be unsuitable for participation in clinical trials by the investigator for other reasons such as alcohol or drug abuse  15) Subjects with pancreatitis or diabetes history or complications  16) Patients with genetic problems such as lactose intolerance and glucose-galactose malabsorption  17) Subjects whose clinical laboratory test results show one of the following or who exhibit abnormal clinical laboratory test values considered to be clinically meaningful by the investigator   - 1. WBC < 3,500/mm^3^   2. Neutrophils < 1,500/mm^3^   3. Hemoglobin < 8.5 g/dL   4. Platelet count < 100,000/mm^3^   5. Serum creatinine > 1.5 × the upper normal limit or 2 mg/dL (whichever is smaller)   6. Total bilirubin > 2 × the upper normal limit   7. AST > 2 × the upper normal limit   8. ALT > 2 × the upper normal limit   9. ALP > 2 × the upper normal limit   10. Glucose: Fasting > 110 mg/dL or postprandial > 200 mg/dL  1. Subjects who are not cooperative with or are unable to follow the clinical trial procedure |
| **Clinical trial period** | - Total research period: 18 months - Subject registration period: 12 months - Test period for each subject: approximately 28 weeks   A. Screening period: within 4 weeks (can be conducted in conjunction with baseline visit)  B. Treatment period: 24 weeks |
| **Clinical trial drug** | 1. **Test drug: Prograf****^®^**   ① Formulation and properties: Light-yellow hard capsule containing white powder (0.5 mg), white hard capsule containing white powder (1 mg)  ② Active ingredient: Tacrolimus  ③ Indication: Chronic rheumatoid arthritis (limited to cases with insufficient efficacy of conventional DMARDs)  ④ Storage method: Airtight container, store at room temperature (1–30 °C)   1. **Reference drug: TNFi**   **Enbrel^®^**  ① Formulation and Appearance: Syringe prefilled with colorless, transparent pale-yellow liquid  ② Active ingredient: Etanercept  ③ Indication: Administration alone or in combination with MTX for active RA in adults with inappropriate responses to DMARDs including MTX  ④ Storage method: sealed container, refrigerated storage (2–8 °C)  **Humira^®^**  ① Formulation and properties: Syringe with an injection needle prefilled with an almost colorless and transparent liquid in a colorless and transparent glass container, or a pen-type container with a prefilled syringe installed inside  ② Active ingredient: Adalimumab  ③ Indication: Treatment of moderate to severe active RA in adults with inappropriate responses to conventional DMARDs including MTX  ④ Storage method: sealed container, refrigerated storage (2–8 °C)  **Remicade^®^**  ① Formulation and properties: vials containing white powder that do not contain diluent  ② Active ingredient: Infliximab  ③ Indications: Symptoms and symptom improvement in the following RA patients:  -Patients with active arthritis with insufficient response to conventional DMARDs, including MTX  -Patients with severe, active, advanced arthritis who have not previously been treated with MTX or other DMARDs  ④ Storage method: sealed container, refrigerated storage (2–8 °C) |
| **Dosage and duration of administration** | 1. **Test drug: Prograf^®^**   Tacrolimus 1 mg is administered orally once a day after dinner.  If it is deemed necessary according to the judgment of the investigator, the amount can be increased up to 3 mg.   1. **Reference drug: TNFi**   The anti-TNF-α agent corresponds to one of the following. Subjects should be administered the same anti-TNF agent as previously used.  (1) Enbrel^®^ 50 mg/syringe, subcutaneous injection, 1 syringes/week (Reduced dose: 1 syringe/2 week)  (2) Humira^®^ 40 mg/syringe, subcutaneous injection, 1 syringe/2 weeks (Reduced dose: 1 syringe/3–4 weeks)  (3) Remicade^®^ 100 mg/vial, intravenous injection, 3–5 mg/kg at 0 weeks, 2 weeks, 6 weeks, and then every 8 weeks until 22 weeks |
| **Methods** | This trial is a multicenter, non-randomized, open, comparative, parallel, 24-week clinical trial. Subjects are RA patients who fulfilled the 2010 ACR/EULAR RA classification criteria and achieved stable LDA with TNFi.  After the subject agrees to participate in the clinical trial, a screening test is performed. The screening test results are evaluated, and subjects that meet the inclusion criteria and do not meet the exclusion criteria are enrolled in the clinical trial. Depending on the selection of the registered subjects, they are included in either the test group (MTX+ Prograf^®^) or the control group (MTX+ TNFi) and receive the investigational drug according to the prescribed dosage of the group.  Subjects in the test group (MTX+ Prograf^®^) receive clinical drugs and take them orally once a day and subjects in the control group (MTX+ TNFi) receive investigational drugs.  Subjects participating in this clinical trial should receive a minimum dose of 7.5 mg/week of MTX prior to the baseline visit and maintain the same dosage and regimen used at the time of study registration, except for reductions due to toxicity during the clinical trial period. Subjects are evaluated for safety and efficacy at baseline and 2, 4, 8, 12, 16, 20, and 24 weeks.  If DAS28 is >2.6 at each visit after baseline and the increase in DAS28 relative to baseline is ≥0.6, this is considered a relapse and treatment failure. The investigator should discontinue administration of the clinical drug and change to another drug. |
| **Concomitant drugs** | Concurrent administration of the following drugs is permitted during this clinical trial period.   \| **Classification** \| **Dosage** \| **Exclusion criteria** \| \| --- \| --- \| --- \| \| Analgesics \| Any dose \| Should not change dosage within 24 h prior to arthritis evaluation \| \| NSAIDS/Cox-2 inhibitor \| Any dose \| Should not change dosage within 14 days prior to arthritis evaluation \| \| Oral corticosteroids \| Maximum dose allowed: ≤10 mg Prednisone equivalent dose/day \| Can be reduced according to domestic guidelines \| \| IM/IV/IA injections of corticosteroids \| Maximum dose allowed:  ≤80 mg  methylprednisolone/ injection \| If necessary, IA injection of corticosteroids within 2 large joints / 1 large joint + 3 small joints / ≤4 small joints between baseline and the 8-week evaluation time point is allowed only once. No additional administration is allowed within 24 weeks. \| |
| **Contrainicated drugs** | Concomitant administration of the following drugs is prohibited during the clinical trial period.   1. IM/IV/IA corticosteroids   *Allowed only once in accordance with the criteria set forth in the acceptable drugs indicated above   1. IA hyaluronic acid 2. DMARDs   sulfasalazine, azathioprine, cyclosporin, cyclophosphamide, hydroxychloroquine, mizoribine, bucillamine, leflunomide, mycophenolate mofetil   1. Concomitant use of potassium-sparing diuretics (spironolactone, triamterene) and excessive intake of potassium 2. Bosentan (Tracleer^®^) |
| **Assessment Outcomes** | 1. **Effectiveness evaluation variables**   ① Primary effectiveness variable  -The proportion of subjects who maintain LDA at 24 weeks*  * LDA defined as DAS28-CRP <3.2  ② Secondary effectiveness variables  i) The proportion of subjects who maintain LDA at 12 weeks  ii) Remission rate at 12 and 24 weeks*  * Remission rate: The proportion of subjects with DAS28-CRP <2.6  iii) Changes in HAQ-DI at 24 weeks compared to baseline  ③ Tertiary effectiveness variables  - Signs and symptoms of disease  i) number of tender joints  ii) number of swollen joints  iii) HAQ-DI (evaluated only at 0-, 12-, and 24-week visit)  iv) Subject's global assessment of arthritis-VAS  v) Investigator's global assessment of arthritis-VAS  vi) CRP, and ESR  vii) Duration of early stiffness  viii) Radiological change by X-ray of hands and feet (Larsen Score)   1. **Safety evaluation variables**   - Adverse reactions  - Vital signs, physical examination, laboratory examination, weight, chest X-ray, pregnancy examination |
| **Statistical analysis** | 1. **Effectiveness evaluation variables**   ① Primary effectiveness variable: LDA at 24 weeks  For each test group, the frequency and percentage of subjects who maintained LDA are presented. In addition, to test differences between the two groups, logistic regression is used.  ② Secondary effectiveness variables  i) LDA at 12 weeks  ii) Remission rate at 12 and 24 weeks  iii) Changes in HAQ-DI at 24 weeks compared to baseline  For (i) and (ii), the frequency and ratio of subjects for each test group are presented. In addition, logistic regression is used to test differences between the two groups.  For (iii), descriptive statistics (frequency, mean, standard deviation, median, minimum, and maximum) are presented for each group, and the differences between baseline and week 24 variables are compared using paired t-tests or the Wilcoxon signed-rank test. In addition, ANCOVA is conducted to test whether there is a difference in the magnitude of change from baseline to week 24 between the two groups.  ③ Tertiary effectiveness variables  a. Signs and symptoms of disease  Continuous data are presented as basic statistics for each visit (frequency, mean, standard deviation, median, minimum, and maximum) for each test group, and paired t-tests or the Wilcoxon signed-rank test is used to compare the differences between baseline values and those of each visit. In addition, an ANCOVA test is conducted to test whether there is a difference in the magnitude of change at each visit from baseline between the two groups. For categorical data, the frequency and ratio of each visit are presented for each test group, and logistic regression is used to test differences between the two groups.   1. **Safety evaluation variables**   a. Adverse reactions  Adverse reactions are classified by body organ using MedDRA, and the frequency and ratio of subjects with adverse reactions in each test group are presented. All adverse reactions are sorted according to their severity, and adverse reactions related to the test drug, serious adverse reactions, and unexpected adverse reactions are distinguished. The chi-square test or Fisher's exact test is performed to test for differences in the incidence of adverse reactions between the two groups.  b. Vital signs, laboratory tests, physical examination, weight, chest X-ray, pregnancy test  Continuous data such as hematology, blood chemistry tests, and vital signs are provided as descriptive statistics (frequency, mean, standard deviation, median, minimum, maximum) for each test group and visit. Categorical variables such as urine tests and physical examinations are presented as the frequency and ratio for each test group and each visit. In addition, the change at week 24 compared to baseline for each group is summarized using a shift table.  All analyses were performed using SAS 9.4 (SAS Institute, Cary, NC, USA) and P-values <0.05 were considered statistically significant. |

**Clinical trial schedule**

|  | **Screening^8)^** | **Baseline** | **Treatment** | | | | | | |
| --- | --- | --- | --- | --- | --- | --- | --- | --- | --- |
| **Weeks (wk)** | **Within 4 wk** | **0** | **2 wk±3 d** | **4 wk±3 d** | **8 wk±5 d** | **12 wk±5 d** | **16 wk±5 d** | **20 wk±5 d** | **24 wk±5 d ^10)^** |
| **Visit** | **1** | **2** | **3** | **4** | **5** | **6** | **7** | **8** | **9** |
| Subject consent | X |  |  |  |  |  |  |  |  |
| Inclusion/Exclusion Criteria | X | X |  |  |  |  |  |  |  |
| Subject basic information | X |  |  |  |  |  |  |  |  |
| Drug administration and medical history investigation | X |  |  |  |  |  |  |  |  |
| Vital signs | X | X | X | X | X | X | X | X | X |
| Weight and height ^1)^ | X | X | X | X | X | X | X | X | X |
| Hematology/blood biochemistry/urine analysis^2)^ | X | X |  |  |  | X |  |  | X |
| Physical examination | X |  |  |  |  | X |  |  | X |
| Chest X-ray^3)^ | X |  |  |  |  |  |  |  |  |
| Hands, feet X-ray |  | X |  |  |  |  |  |  | X |
| Patient's assessment^4)^ | X | X | X | X | X | X | X | X | X |
| Physician's assessment^5)^ | X | X | X | X | X | X | X | X | X |
| Pregnancy test^6)^ | X |  |  |  |  |  |  |  | X |
| Concomitant drugs^7)^ | X | X | X | X | X | X | X | X | X |
| Adverse reaction |  | X | X | X | X | X | X | X | X |
| Prescription of clinical trial drugs |  | X^9)^ | X | X | X | X | X | X |  |

1. Body weight is measured at every visit, but height is measured only at the time of screening.
2. Laboratory tests

- Hematology: WBC, RBC, Hb, Hct, ESR, platelets, WBC differential count
- Serum chemistry: Na^+^, K^+^, Cl^-^, bicarbonate (total CO_2_), Ca^2+^, P, BUN, CPK, ALT, AST, glucose, total bilirubin, total cholesterol, HDL cholesterol, triglycerides, LDL cholesterol, γ-GTP, uric acid, total protein, albumin, ALP, creatinine, CRP, LDH
- Urine analysis: pH, protein, glucose, blood, urine microscopy (RBC, WBC, epithelial cells, casts, bacteria, crystals)

1. Patients with no chest X-ray results within 3 months prior to the baseline visit or with respiratory symptoms.
2. HAQ-DI, pain assessment, overall assessment of arthritis, duration of morning stiffness. In the case of HAQ-DI, it is only performed at 0, 12, and 24 weeks.
3. Number of tender joints and swollen joints, evaluation of the physician's global assessment
4. A pregnancy test (serum or urine) is performed at the screening visit and at the 24^th^ week/dropout visit.
5. Subjects should not take the following concomitant drugs within 4 weeks of the baseline visit: IM/IV/IA corticosteroids, IA hyaluronic acid, DMARDs (sulfasalazine, azathioprine, cyclosporin, cyclophosphamide, hydroxychloroquine, mizoribine, bucillamine, leflunomide, mycophenolate mofetil, tacrolimus)
6. Data acquired by the same institution within 4 weeks before screening can be used for screening evaluation and can be integrated with the baseline data.
7. Administer the investigational drug.
8. In case of early termination, the same inspection and evaluation as the termination visit shall be performed.

**Table 1.1 Subject disposition (Screened Set)**

|  | **MTX + Prograf** | **MTX + Anti TNF-α** | **Total** |
| --- | --- | --- | --- |
| **Screening** |  |  | 130 |
| **Screening Failure** |  |  | 10 |
| **Enrolled, n(%)** | 35 | 85 | 120 |
| Treated | 34 | 84 | 118 |
| Non-Treated | 1 | 1 | 2 |
| **Complated Status, n(%)** |  |  |  |
| Completion | 26 | 78 | 104 |
| Discontinuation | 9 | 7 | 16 |
| **Primary Reason for Discontinuation** |  |  |  |
| Voluntary suspension | 3 | 4 | 7 |
| Safety reasons | 4 | 0 | 4 |
| Violation of clinical trial plan (violation of inclusion/exclusion criteria, administration of contraindicated medications, etc.) | 1 | 0 | 1 |
| Patients who participated in violation of the inclusion/exclusion criteria | 0 | 3 | 3 |
| DAS28 > 2.6 at every visit after the baseline, and DAS28 increases over the baseline is greater than 0.6 | 0 | 0 | 0 |
| Other | 1 | 0 | 1 |

**Table 1.2 Analysis Sets (Enrolled Set)**

|  | **MTX + Prograf** | **MTX + Anti TNF-α** | **Total** |
| --- | --- | --- | --- |
| **Full Analysis Set** | 34 | 80 | 114 |
| **Reason for Exclusion from Full Analysis Set** |  |  |  |
| Other | 0 | 4 | 4 |
| Not treated | 1 | 1 | 2 |
| **Per-Protocol Set** | 22 | 62 | 84 |
| **Reason for Exclusion from Per-Protocol Set** |  |  |  |
| Violation of inclusion criteria | 0 | 11 | 11 |
| Violation of exclusion criteria | 2 | 3 | 5 |
| Violation of clinical trial medication | 4 | 0 | 4 |
| Other | 0 | 3 | 3 |
| Not treated | 1 | 1 | 2 |
| Violation of inclusion criteria and drug-related violations for clinical trials | 2 | 0 | 2 |
| Violation of inclusion/exclusion criteria | 0 | 1 | 1 |
| Violation of exclusion criteria, Other | 0 | 1 | 1 |
| Drop out | 0 | 1 | 1 |
| **Safety Set** | 34 | 84 | 118 |
| **Reason for Exclusion from Safety Set** |  |  |  |
| Not treated | 1 | 1 | 2 |

**Table 1.3 Major Protocol Deviation (Enrolled Set)**

|  | **MTX + Prograf**  **(n = 35)** | **MTX + Anti TNF-α**  **(n = 85)** | **Total**  **(n = 120)** |
| --- | --- | --- | --- |
| **Subjects with Major Protocol Deviation** | 10 | 22 | 32 |
| Violation of inclusion criteria | 2(5.71) [2] | 12(14.12) [12] | 14(11.67) [14] |
| Violation of exclusion criteria | 2(5.71) [2] | 5(5.88) [5] | 7(5.83) [7] |
| **Drug-related violations for clinical trials** | 6(17.14) [6] | 0 | 6(5.00) [6] |
| Other | 0 | 4(4.71) [4] | 4(3.33) [4] |
| Drop out | 0 | 1(1.18) [1] | 1(0.83) [1] |

Major protocol deviation are displayed as 'number of subjects(percentage of subjects) [number of events]'.

**Table 1.4 Medical History by SOC and PT (Full Analysis Set)**

|  | **MTX + Prograf**  **(n = 35)** | **MTX + Anti TNF-α**  **(n = 85)** | **Total**  **(n = 120)** |
| --- | --- | --- | --- |
| **Subjects with Medical History** | 3(8.82) [3] | 5(6.25) [6] | 8(7.02) [9] |
| P-value [1] |  |  | 0.6937 (f) |
| **Infections and infestations** | 0 | 3(3.75) [3] | 3(2.63) [3] |
| Herpes zoster | 0 | 1(1.25) [1] | 1(0.88) [1] |
| Latent tuberculosis | 0 | 1(1.25) [1] | 1(0.88) [1] |
| Sinusitis | 0 | 1(1.25) [1] | 1(0.88) [1] |
| **Surgical and medical procedures** | 1(2.94) [1] | 1(1.25) [2] | 2(1.75) [3] |
| Intervertebral disc operation | 1(2.94) [1] | 1(1.25) [2] | 2(1.75) [3] |
| **General disorders and administration site conditions** | 1(2.94) [1] | 1(1.25) [1] | 2(1.75) [2] |
| Chest discomfort | 0 | 1(1.25) [1] | 1(0.88) [1] |
| Pelvic mass | 1(2.94) [1] | 0 | 1(0.88) [1] |
| **Respiratory, thoracic and mediastinal disorders** | 1(2.94) [1] | 0 | 1(0.88) [1] |
| Rhinitis allergic | 1(2.94) [1] | 0 | 1(0.88) [1] |

[1] Testing for difference between treatment groups (chi-square test (c) or Fisher's exact test (f)).

Note: Denominator of percentage is the number of subjects in the each treatment group.

Medical histories are displayed as 'number of subjects(percentage of subjects) [number of event]'

**Table 1.5 Treatment Compliance**

|  | **MTX + Prograf (N=34)** |
| --- | --- |
| **Compliance at Week 8** |  |
| n | 34 |
| Mean(SD) | 93.87(13.07) |
| Median | 100.00 |
| Min, Max | 40.00, 103.57 |
| **Compliance at Week 16** |  |
| n | 27 |
| Mean(SD) | 95.83(10.14) |
| Median | 100.00 |
| Min, Max | 50.00, 100.00 |
| **Compliance at Week 24** |  |
| n | 27 |
| Mean(SD) | 86.77(23.77) |
| Median | 98.21 |
| Min, Max | 5.36, 100.00 |
| **Overall Compliance** |  |
| n | 34 |
| Mean(SD) | 89.67(16.21) |
| Median | 98.20 |
| Min, Max | 40.00, 101.33 |

**Table 2.1 Summary of Morning Stiffness (Per-Protocol Set)**

|  | **MTX + Prograf (N=34)** | **MTX + Anti TNF-α (N=80)** |
| --- | --- | --- |
| **Baseline** |  |  |
| n | 34 | 80 |
| Mean(SD) | 7.65(12.97) | 14.13(48.46) |
| Median | 0.00 | 0.00 |
| Min, Max | 0.00, 60.00 | 0.00, 420.00 |
| **Week 2** |  |  |
| n | 32 |  |
| Mean(SD) | 11.84(17.79) |  |
| Median | 3.50 |  |
| Min, Max | 0.00, 70.00 |  |
| **Change from Baseline at Week 2** |  |  |
| n | 32 |  |
| Mean(SD) | 3.72(19.66) |  |
| Median | 0.00 |  |
| Min, Max | -50.00, 70.00 |  |
| P-value [1] | 0.3957 (w) |  |
| **Week 4** |  |  |
| n | 32 |  |
| Mean(SD) | 15.38(27.44) |  |
| Median | 5.00 |  |
| Min, Max | 0.00, 120.00 |  |
| **Change from Baseline at Week 4** |  |  |
| n | 32 |  |
| Mean(SD) | 7.25(21.22) |  |
| Median | 0.00 |  |
| Min, Max | -30.00, 65.00 |  |
| P-value [1] | 0.1241 (w) |  |
| **Week 8** |  |  |
| n | 32 | 79 |
| Mean(SD) | 20.53(54.28) | 18.92(50.03) |
| Median | 3.50 | 1.00 |
| Min, Max | 0.00, 300.00 | 0.00, 420.00 |
| **Change from Baseline at Week 8** |  |  |
| n | 32 | 79 |
| Mean(SD) | 12.41(51.74) | 5.00(19.21) |
| Median | 0.00 | 0.00 |
| Min, Max | -30.00, 280.00 | -30.00, 60.00 |
| P-value [1] | 0.3700 (w) | 0.0618 (w) |
| **ANCOVA Result at Week 8 [2]** |  |  |
| LS Mean(SE) | 12.23 (5.69) | 5.07 (3.62) |
| LS Mean Difference(SE) | 7.16 (6.74) |  |
| 95% Confidence Interval for Difference | [-6.20, 20.53] |  |
| P-value | 0.2905 (a) |  |
| **Week 16** |  |  |
| n | 32 | 79 |
| Mean(SD) | 30.00(80.16) | 12.53(19.04) |
| Median | 7.50 | 5.00 |
| Min, Max | 0.00, 360.00 | 0.00, 70.00 |
| **Change from Baseline at Week 16** |  |  |
| n | 32 | 79 |
| Mean(SD) | 21.88(80.34) | -1.39(50.13) |
| Median | 0.00 | 0.00 |
| Min, Max | -30.00, 360.00 | -420.00, 65.00 |
| P-value [1] | 0.2994 (w) | 0.0778 (w) |
| **ANCOVA Result at Week 16 [2]** |  |  |
| LS Mean(SE) | 17.99 (8.11) | 0.18 (5.16) |
| LS Mean Difference(SE) | 17.80 (9.62) |  |
| 95% Confidence Interval for Difference | [-1.27, 36.88] |  |
| P-value | 0.0670 (a) |  |
| **Week 24** |  |  |
| n | 34 | 79 |
| Mean(SD) | 60.88(251.50) | 14.63(26.04) |
| Median | 0.00 | 5.00 |
| Min, Max | 0.00, 1440.00 | 0.00, 120.00 |
| **Change from Baseline at Week 24** |  |  |
| n | 34 | 79 |
| Mean(SD) | 53.24(250.05) | 0.71(47.75) |
| Median | 0.00 | 0.00 |
| Min, Max | -60.00, 1420.00 | -360.00, 119.00 |
| P-value [1] | 0.6004 (w) | 0.2284 (w) |
| **ANCOVA Result at Week 24 [2]** |  |  |
| LS Mean(SE) | 49.88 (23.90) | 2.15 (15.67) |
| LS Mean Difference(SE) | 47.73 (28.61) |  |
| 95% Confidence Interval for Difference | [-8.97, 104.43] |  |
| P-value | 0.0981 (a) |  |

SD = standard deviation, Min = minimum, Max = maximum, LS means = least square means, SE = standard error, CI = confidence interval.

[1] Testing for change within-treatment groups (paired t-test (p) or Wilcoxon signed rank test (w)).

[2] Testing for difference between treatment (ANCOVA model with treatment group as a factor and baseline value as a covariate).

Note: Baseline visit is Visit 2 (Day 0).

**Table 2.2 Summary of Larsen score (Per-Protocol Set)**

|  | **MTX + Prograf (N=22)** | **MTX + Anti TNF-α (N=62)** |
| --- | --- | --- |
| **Baseline** |  |  |
| n | 21 | 60 |
| Mean(SD) | 11.62(19.78) | 8.57(15.56) |
| Median | 3.00 | 2.00 |
| Min, Max | 0.00, 85.00 | 0.00, 86.00 |
| **Week 24** |  |  |
| n | 19 | 60 |
| Mean(SD) | 12.05(21.40) | 8.75(15.89) |
| Median | 3.00 | 2.00 |
| Min, Max | 0.00, 85.00 | 0.00, 89.00 |
| **Change from Baseline at Week 24** |  |  |
| n | 19 | 60 |
| Mean(SD) | 0.11(1.73) | 0.18(1.67) |
| Median | 0.00 | 0.00 |
| Min, Max | -2.00, 6.00 | -8.00, 5.00 |
| P-value [1] | 0.7266 (w) | 0.2056 (w) |
| **ANCOVA Result at Week 24 [2]** |  |  |
| LS Mean(SE) | 0.05 (0.38) | 0.20 (0.21) |
| LS Mean Difference(SE) | -0.15 (0.44) |  |
| 95% Confidence Interval for Difference | [-1.02, 0.72] |  |
| P-value | 0.7302 (a) |  |

DAS28 = Disease Activity Score of 28, SD = standard deviation, Min = minimum, Max = maximum, LS means = least square means, SE = standard error, CI = confidence interval.

[1] Testing for change within-treatment groups (paired t-test (p) or Wilcoxon signed rank test (w)).

[2] Testing for difference between treatment (ANCOVA model with treatment group as a factor and baseline value as a covariate).

Note: Baseline visit is Visit 2 (Day 0).

|  | **MTX + Prograf (N=34)** | **MTX + Anti TNF-α (N=84)** | | |
| --- | --- | --- | --- | --- |
|  | **Prograf** | **Enbrel** | **Humira** | **Remicade** |
| **Total Dosage (mg)** |  |  |  |  |
| n | 34 | 41 | 38 | 5 |
| Mean(SD) | 176.34(163.23) | 1084.15(261.17) | 454.74(142.39) | 640.00(260.77) |
| Median | 160.00 | 1200.00 | 480.00 | 800.00 |
| Min, Max | 4.00, 911.00 | 50.00, 1350.00 | 40.00, 880.00 | 200.00, 800.00 |

**Table 3.1 Extent of Exposure (Safety Set)**

SD = standard deviation, Min = minimum, Max = maximum.

Note: Total dosage (mg) = Number of actually taken days (days) * actually taken dose (mg)

**Table 3.2 Overall Summary of TEAE (Safety Set)**

|  | **MTX + Prograf (N=34)** | **MTX + Anti TNF-α (N=84)** | **Total (N=118)** |
| --- | --- | --- | --- |
| **Subjects with TEAEs** | 16(47.06) [17] | 19(22.62) [25] | 35(29.66) [42] |
| 95% Confidence Interval | [30.28, 63.84] | [13.67, 31.57] | [21.42, 37.90] |
| P-value [1] |  |  | 0.0085 (c) |
| **Severity** |  |  |  |
| Mild | 12 | 23 | 35 |
| Moderate | 3 | 2 | 5 |
| Severe | 2 | 0 | 2 |
| **Relationship to IP** |  |  |  |
| Not clearly relevant | 5 | 10 | 15 |
| Not relevant | 5 | 8 | 13 |
| Possibly not relevant | 4 | 7 | 11 |
| Possibly relevant | 3 | 0 | 3 |
| Clearly relevant | 0 | 0 | 0 |
| Unknown | 0 | 0 | 0 |
| **Subjects with Serious TEAEs** | 1(2.94) [1] | 0 | 1(0.85) [1] |
| Exact 95% Confidence Interval | [0.07, 15.33] | [0.00, 4.30] | [0.02, 4.63] |
| P-value [1] |  |  | 0.2881 (f) |
| **Subjects with TEAEs Leading to Permanent Discontinuation** | 6(17.65) [6] | 0 | 6(5.08) [6] |
| Exact 95% Confidence Interval | [6.76, 34.53] | [0.00, 4.30] | [1.89, 10.74] |
| P-value [1] |  |  | 0.0004 (f) |
| **Subjects with TEAEs Leading to Death** | 0 | 0 | 0 |
| Exact 95% Confidence Interval | [0.00, 10.28] | [0.00, 4.30] | [0.00, 3.08] |
| P-value [1] |  |  | NC |

TEAEs = treatment-emergent adverse events, SOC = system organ class, PT = preferred term, NC: Not calculated.

[1] Testing for difference between treatment groups (chi-square test (c) or Fisher's exact test (f)).

Note: Denominator of percentage is the number of subjects in the each treatment group.

TEAEs are displayed as 'number of subjects(percentage of subjects) [number of events]'.

**Table 3.3 Overall Summary of ADR (Safety Set)**

|  | **MTX + Prograf (N=34)** | **MTX + Anti TNF-α (N=84)** | **Total (N=118)** |
| --- | --- | --- | --- |
| **Subjects with ADRs** | 7(20.59) [7] | 6(7.14) [7] | 13(11.02) [14] |
| Exact 95% Confidence Interval | [8.70, 37.90] | [2.67, 14.90] | [6.00, 18.10] |
| P-value [1] |  |  | 0.0501 (f) |
| **Severity** |  |  |  |
| Mild | 4 | 6 | 10 |
| Moderate | 1 | 1 | 2 |
| Severe | 2 | 0 | 2 |
| **Relationship to IP** |  |  |  |
| Not clearly relevant | 0 | 0 | 0 |
| Not relevant | 0 | 0 | 0 |
| Possibly not relevant | 4 | 7 | 11 |
| Possibly relevant | 3 | 0 | 3 |
| Clearly relevant | 0 | 0 | 0 |
| Unknown | 0 | 0 | 0 |
| **Subjects with Serious ADRs** | 1(2.94) [1] | 0 | 1(0.85) [1] |
| Exact 95% Confidence Interval | [0.07, 15.33] | [0.00, 4.30] | [0.02, 4.63] |
| P-value [1] |  |  | 0.2881 (f) |
| **Subjects with ADRs Leading to Permanent Discontinuation** | 4(11.76) [4] | 0 | 4(3.39) [4] |
| Exact 95% Confidence Interval | [3.30, 27.45] | [0.00, 4.30] | [0.93, 8.45] |
| P-value [1] |  |  | 0.0060 (f) |
| **Subjects with ADRs Leading to Death** | 0 | 0 | 0 |
| Exact 95% Confidence Interval | [0.00, 10.28] | [0.00, 4.30] | [0.00, 3.08] |
| P-value [1] |  |  | NC |

ADRs = adverse drug reactions, SOC = system organ class, PT = preferred term, NC: Not calculated.

[1] Testing for difference between treatment groups (chi-square test (c) or Fisher's exact test (f)).

Note: Denominator of percentage is the number of subjects in the each treatment group.

ADRs are displayed as 'number of subjects(percentage of subjects) [number of events]'.

**Table 3.4 Incidence of TEAEs by SOC and PT (Safety Set)**

|  | **MTX + Prograf (N=34)** | **MTX + Anti TNF-α (N=84)** | **Total (N=118)** |
| --- | --- | --- | --- |
| **Subjects with TEAEs** | 16(47.06) [17] | 19(22.62) [25] | 35(29.66) [42] |
| **Infections and infestations** | 2(5.88) [2] | 9(10.71) [10] | 11(9.32) [12] |
| Viral upper respiratory tract infection | 0 | 5(5.95) [5] | 5(4.24) [5] |
| Upper respiratory tract infection | 1(2.94) [1] | 3(3.57) [3] | 4(3.39) [4] |
| Cystitis | 0 | 1(1.19) [1] | 1(0.85) [1] |
| Disseminated tuberculosis | 1(2.94) [1] | 0 | 1(0.85) [1] |
| Oral herpes | 0 | 1(1.19) [1] | 1(0.85) [1] |
| **Musculoskeletal and connective tissue disorders** | 7(20.59) [8] | 2(2.38) [2] | 9(7.63) [10] |
| Arthralgia | 2(5.88) [2] | 0 | 2(1.69) [2] |
| Arthritis | 1(2.94) [1] | 0 | 1(0.85) [1] |
| Axillary mass | 1(2.94) [1] | 0 | 1(0.85) [1] |
| Joint swelling | 1(2.94) [1] | 0 | 1(0.85) [1] |
| Musculoskeletal pain | 1(2.94) [1] | 0 | 1(0.85) [1] |
| Myalgia | 1(2.94) [1] | 0 | 1(0.85) [1] |
| Neck pain | 1(2.94) [1] | 0 | 1(0.85) [1] |
| Rheumatoid arthritis | 0 | 1(1.19) [1] | 1(0.85) [1] |
| Scoliosis | 0 | 1(1.19) [1] | 1(0.85) [1] |
| **Gastrointestinal disorders** | 4(11.76) [4] | 2(2.38) [2] | 6(5.08) [6] |
| Abdominal pain upper | 2(5.88) [2] | 0 | 2(1.69) [2] |
| Abdominal discomfort | 0 | 1(1.19) [1] | 1(0.85) [1] |
| Abdominal pain | 1(2.94) [1] | 0 | 1(0.85) [1] |
| Abdominal pain lower | 1(2.94) [1] | 0 | 1(0.85) [1] |
| Gastric polyps | 0 | 1(1.19) [1] | 1(0.85) [1] |
| **Nervous system disorders** | 2(5.88) [2] | 1(1.19) [1] | 3(2.54) [3] |
| Headache | 1(2.94) [1] | 1(1.19) [1] | 2(1.69) [2] |
| Lethargy | 1(2.94) [1] | 0 | 1(0.85) [1] |
| **Respiratory, thoracic and mediastinal disorders** | 0 | 3(3.57) [3] | 3(2.54) [3] |
| Cough | 0 | 2(2.38) [2] | 2(1.69) [2] |
| Rhinitis allergic | 0 | 1(1.19) [1] | 1(0.85) [1] |
| **Metabolism and nutrition disorders** | 0 | 2(2.38) [2] | 2(1.69) [2] |
| Dyslipidaemia | 0 | 1(1.19) [1] | 1(0.85) [1] |
| Hypercholesterolaemia | 0 | 1(1.19) [1] | 1(0.85) [1] |
| **Skin and subcutaneous tissue disorders** | 0 | 2(2.38) [2] | 2(1.69) [2] |
| Alopecia | 0 | 1(1.19) [1] | 1(0.85) [1] |
| Rash | 0 | 1(1.19) [1] | 1(0.85) [1] |
| **Blood and lymphatic system disorders** | 0 | 1(1.19) [1] | 1(0.85) [1] |
| Lymphadenopathy | 0 | 1(1.19) [1] | 1(0.85) [1] |
| **Injury, poisoning and procedural complications** | 0 | 1(1.19) [1] | 1(0.85) [1] |
| Hand fracture | 0 | 1(1.19) [1] | 1(0.85) [1] |
| **Psychiatric disorders** | 1(2.94) [1] | 0 | 1(0.85) [1] |
| Insomnia | 1(2.94) [1] | 0 | 1(0.85) [1] |
| **Surgical and medical procedures** | 0 | 1(1.19) [1] | 1(0.85) [1] |
| Haemorrhoid operation | 0 | 1(1.19) [1] | 1(0.85) [1] |

TEAEs = treatment-emergent adverse events, SOC = system organ class, PT = preferred term

Note: Denominator of percentage is the number of subjects in the each treatment group.

TEAEs are displayed as 'number of subjects(percentage of subjects) [number of events]'.

**Table 3.5 Incidence of ADRs by SOC and PT (Safety set)**

|  | **MTX + Prograf (N=34)** | **MTX + Anti TNF-α (N=84)** | **Total (N=118)** |
| --- | --- | --- | --- |
| **Subjects with ADRs** | 7(20.59) [7] | 6(7.14) [7] | 13(11.02) [14] |
| **Infections and infestations** | 1(2.94) [1] | 4(4.76) [5] | 5(4.24) [6] |
| Viral upper respiratory tract infection | 0 | 3(3.57) [3] | 3(2.54) [3] |
| Disseminated tuberculosis | 1(2.94) [1] | 0 | 1(0.85) [1] |
| Oral herpes | 0 | 1(1.19) [1] | 1(0.85) [1] |
| Upper respiratory tract infection | 0 | 1(1.19) [1] | 1(0.85) [1] |
| **Gastrointestinal disorders** | 4(11.76) [4] | 0 | 4(3.39) [4] |
| Abdominal pain upper | 2(5.88) [2] | 0 | 2(1.69) [2] |
| Abdominal pain | 1(2.94) [1] | 0 | 1(0.85) [1] |
| Abdominal pain lower | 1(2.94) [1] | 0 | 1(0.85) [1] |
| **Musculoskeletal and connective tissue disorders** | 1(2.94) [1] | 1(1.19) [1] | 2(1.69) [2] |
| Arthralgia | 1(2.94) [1] | 0 | 1(0.85) [1] |
| Rheumatoid arthritis | 0 | 1(1.19) [1] | 1(0.85) [1] |
| **Blood and lymphatic system disorders** | 0 | 1(1.19) [1] | 1(0.85) [1] |
| Lymphadenopathy | 0 | 1(1.19) [1] | 1(0.85) [1] |
| **Nervous system disorders** | 1(2.94) [1] | 0 | 1(0.85) [1] |
| Headache | 1(2.94) [1] | 0 | 1(0.85) [1] |

ADRs = adverse drug reactions, SOC = system organ class, PT = preferred term

Note: Denominator of percentage is the number of subjects in the each treatment group.

ADRs are displayed as 'number of subjects(percentage of subjects) [number of events]'.

**Table 3.6 Incidence of TEAE by Maximum Severity, SOC and PT (Safety Set)**

|  | **MTX + Prograf (N=34)** | | | **MTX + Anti TNF-α (N=84)** | | | **Total (N=118)** | | |
| --- | --- | --- | --- | --- | --- | --- | --- | --- | --- |
|  | **Mild** | **Moderate** | **Severe** | **Mild** | **Moderate** | **Severe** | **Mild** | **Moderate** | **Severe** |
| **Infections and infestations** | 1(2.94) | 0 | 1(2.94) | 8(9.52) | 1(1.19) | 0 | 9(7.63) | 1(0.85) | 1(0.85) |
| Viral upper respiratory tract infection | 0 | 0 | 0 | 4(4.76) | 1(1.19) | 0 | 4(3.39) | 1(0.85) | 0 |
| Upper respiratory tract infection | 1(2.94) | 0 | 0 | 3(3.57) | 0 | 0 | 4(3.39) | 0 | 0 |
| Cystitis | 0 | 0 | 0 | 1(1.19) | 0 | 0 | 1(0.85) | 0 | 0 |
| Disseminated tuberculosis | 0 | 0 | 1(2.94) | 0 | 0 | 0 | 0 | 0 | 1(0.85) |
| Oral herpes | 0 | 0 | 0 | 1(1.19) | 0 | 0 | 1(0.85) | 0 | 0 |
| **Musculoskeletal and connective tissue disorders** | 5(14.71) | 2(5.88) | 0 | 2(2.38) | 0 | 0 | 7(5.93) | 2(1.69) | 0 |
| Arthralgia | 2(5.88) | 0 | 0 | 0 | 0 | 0 | 2(1.69) | 0 | 0 |
| Arthritis | 0 | 1(2.94) | 0 | 0 | 0 | 0 | 0 | 1(0.85) | 0 |
| Axillary mass | 1(2.94) | 0 | 0 | 0 | 0 | 0 | 1(0.85) | 0 | 0 |
| Joint swelling | 0 | 1(2.94) | 0 | 0 | 0 | 0 | 0 | 1(0.85) | 0 |
| Musculoskeletal pain | 1(2.94) | 0 | 0 | 0 | 0 | 0 | 1(0.85) | 0 | 0 |
| Myalgia | 1(2.94) | 0 | 0 | 0 | 0 | 0 | 1(0.85) | 0 | 0 |
| Neck pain | 1(2.94) | 0 | 0 | 0 | 0 | 0 | 1(0.85) | 0 | 0 |
| Rheumatoid arthritis | 0 | 0 | 0 | 1(1.19) | 0 | 0 | 1(0.85) | 0 | 0 |
| Scoliosis | 0 | 0 | 0 | 1(1.19) | 0 | 0 | 1(0.85) | 0 | 0 |
| **Gastrointestinal disorders** | 2(5.88) | 1(2.94) | 1(2.94) | 2(2.38) | 0 | 0 | 4(3.39) | 1(0.85) | 1(0.85) |
| Abdominal pain upper | 1(2.94) | 1(2.94) | 0 | 0 | 0 | 0 | 1(0.85) | 1(0.85) | 0 |
| Abdominal discomfort | 0 | 0 | 0 | 1(1.19) | 0 | 0 | 1(0.85) | 0 | 0 |
| Abdominal pain | 0 | 0 | 1(2.94) | 0 | 0 | 0 | 0 | 0 | 1(0.85) |
| Abdominal pain lower | 1(2.94) | 0 | 0 | 0 | 0 | 0 | 1(0.85) | 0 | 0 |
| Gastric polyps | 0 | 0 | 0 | 1(1.19) | 0 | 0 | 1(0.85) | 0 | 0 |
| **Nervous system disorders** | 2(5.88) | 0 | 0 | 1(1.19) | 0 | 0 | 3(2.54) | 0 | 0 |
| Headache | 1(2.94) | 0 | 0 | 1(1.19) | 0 | 0 | 2(1.69) | 0 | 0 |
| Lethargy | 1(2.94) | 0 | 0 | 0 | 0 | 0 | 1(0.85) | 0 | 0 |
| **Respiratory, thoracic and mediastinal disorders** | 0 | 0 | 0 | 3(3.57) | 0 | 0 | 3(2.54) | 0 | 0 |
| Cough | 0 | 0 | 0 | 2(2.38) | 0 | 0 | 2(1.69) | 0 | 0 |
| Rhinitis allergic | 0 | 0 | 0 | 1(1.19) | 0 | 0 | 1(0.85) | 0 | 0 |
| **Metabolism and nutrition disorders** | 0 | 0 | 0 | 1(1.19) | 1(1.19) | 0 | 1(0.85) | 1(0.85) | 0 |
| Dyslipidaemia | 0 | 0 | 0 | 0 | 1(1.19) | 0 | 0 | 1(0.85) | 0 |
| Hypercholesterolaemia | 0 | 0 | 0 | 1(1.19) | 0 | 0 | 1(0.85) | 0 | 0 |
| **Skin and subcutaneous tissue disorders** | 0 | 0 | 0 | 2(2.38) | 0 | 0 | 2(1.69) | 0 | 0 |
| Alopecia | 0 | 0 | 0 | 1(1.19) | 0 | 0 | 1(0.85) | 0 | 0 |
| Rash | 0 | 0 | 0 | 1(1.19) | 0 | 0 | 1(0.85) | 0 | 0 |
| **Blood and lymphatic system disorders** | 0 | 0 | 0 | 1(1.19) | 0 | 0 | 1(0.85) | 0 | 0 |
| Lymphadenopathy | 0 | 0 | 0 | 1(1.19) | 0 | 0 | 1(0.85) | 0 | 0 |
| **Injury, poisoning and procedural complications** | 0 | 0 | 0 | 1(1.19) | 0 | 0 | 1(0.85) | 0 | 0 |
| Hand fracture | 0 | 0 | 0 | 1(1.19) | 0 | 0 | 1(0.85) | 0 | 0 |
| **Psychiatric disorders** | 1(2.94) | 0 | 0 | 0 | 0 | 0 | 1(0.85) | 0 | 0 |
| Insomnia | 1(2.94) | 0 | 0 | 0 | 0 | 0 | 1(0.85) | 0 | 0 |
| **Surgical and medical procedures** | 0 | 0 | 0 | 1(1.19) | 0 | 0 | 1(0.85) | 0 | 0 |
| Haemorrhoid operation | 0 | 0 | 0 | 1(1.19) | 0 | 0 | 1(0.85) | 0 | 0 |

TEAEs = treatment-emergent adverse events, SOC = system organ class, PT = preferred term

Note: Denominator of percentage is the number of subjects in the each treatment group.

TEAEs are displayed as number of subjects(percentage of subjects).

Where one subject experiences the same preferred term more than once, the preferred term is counted only once in the most severe category.

**Table 3.7 Incidence of ADRs by Maximum Severity, SOC and PT (Safety Set)**

|  | **MTX + Prograf (N=34)** | | | **MTX + Anti TNF-α (N=84)** | | | **Total (N=118)** | | |
| --- | --- | --- | --- | --- | --- | --- | --- | --- | --- |
|  | **Mild** | **Moderate** | **Severe** | **Mild** | **Moderate** | **Severe** | **Mild** | **Moderate** | **Severe** |
| **Infections and infestations** | 0 | 0 | 1(2.94) | 3(3.57) | 1(1.19) | 0 | 3(2.54) | 1(0.85) | 1(0.85) |
| Viral upper respiratory tract infection | 0 | 0 | 0 | 2(2.38) | 1(1.19) | 0 | 2(1.69) | 1(0.85) | 0 |
| Disseminated tuberculosis | 0 | 0 | 1(2.94) | 0 | 0 | 0 | 0 | 0 | 1(0.85) |
| Oral herpes | 0 | 0 | 0 | 1(1.19) | 0 | 0 | 1(0.85) | 0 | 0 |
| Upper respiratory tract infection | 0 | 0 | 0 | 1(1.19) | 0 | 0 | 1(0.85) | 0 | 0 |
| **Gastrointestinal disorders** | 2(5.88) | 1(2.94) | 1(2.94) | 0 | 0 | 0 | 2(1.69) | 1(0.85) | 1(0.85) |
| Abdominal pain upper | 1(2.94) | 1(2.94) | 0 | 0 | 0 | 0 | 1(0.85) | 1(0.85) | 0 |
| Abdominal pain | 0 | 0 | 1(2.94) | 0 | 0 | 0 | 0 | 0 | 1(0.85) |
| Abdominal pain lower | 1(2.94) | 0 | 0 | 0 | 0 | 0 | 1(0.85) | 0 | 0 |
| **Musculoskeletal and connective tissue disorders** | 1(2.94) | 0 | 0 | 1(1.19) | 0 | 0 | 2(1.69) | 0 | 0 |
| Arthralgia | 1(2.94) | 0 | 0 | 0 | 0 | 0 | 1(0.85) | 0 | 0 |
| Rheumatoid arthritis | 0 | 0 | 0 | 1(1.19) | 0 | 0 | 1(0.85) | 0 | 0 |
| **Blood and lymphatic system disorders** | 0 | 0 | 0 | 1(1.19) | 0 | 0 | 1(0.85) | 0 | 0 |
| Lymphadenopathy | 0 | 0 | 0 | 1(1.19) | 0 | 0 | 1(0.85) | 0 | 0 |
| **Nervous system disorders** | 1(2.94) | 0 | 0 | 0 | 0 | 0 | 1(0.85) | 0 | 0 |
| Headache | 1(2.94) | 0 | 0 | 0 | 0 | 0 | 1(0.85) | 0 | 0 |

ADRs = adverse drug reactions, SOC = system organ class, PT = preferred term

Note: Denominator of percentage is the number of subjects in the each treatment group.

ADRs are displayed as number of subjects(percentage of subjects).

Where one subject experiences the same preferred term more than once, the preferred term is counted only once in the most severe category.

**Table 3.8 Incidence of TEAE by Maximum Relationship, SOC and PT (Safety Set)**

|  | **MTX + Prograf (N=34)** | | **MTX + Anti TNF-α (N=84)** | | **Total (N=118)** | |
| --- | --- | --- | --- | --- | --- | --- |
|  | **Related** | **Not related** | **Related** | **Not related** | **Related** | **Not related** |
| **Infections and infestations** | 1(2.94) | 1(2.94) | 4(4.76) | 5(5.95) | 5(4.24) | 6(5.08) |
| Viral upper respiratory tract infection | 0 | 0 | 3(3.57) | 2(2.38) | 3(2.54) | 2(1.69) |
| Upper respiratory tract infection | 0 | 1(2.94) | 1(1.19) | 2(2.38) | 1(0.85) | 3(2.54) |
| Cystitis | 0 | 0 | 0 | 1(1.19) | 0 | 1(0.85) |
| Disseminated tuberculosis | 1(2.94) | 0 | 0 | 0 | 1(0.85) | 0 |
| Oral herpes | 0 | 0 | 1(1.19) | 0 | 1(0.85) | 0 |
| **Musculoskeletal and connective tissue disorders** | 1(2.94) | 6(17.65) | 1(1.19) | 1(1.19) | 2(1.69) | 7(5.93) |
| Arthralgia | 1(2.94) | 1(2.94) | 0 | 0 | 1(0.85) | 1(0.85) |
| Arthritis | 0 | 1(2.94) | 0 | 0 | 0 | 1(0.85) |
| Axillary mass | 0 | 1(2.94) | 0 | 0 | 0 | 1(0.85) |
| Joint swelling | 0 | 1(2.94) | 0 | 0 | 0 | 1(0.85) |
| Musculoskeletal pain | 0 | 1(2.94) | 0 | 0 | 0 | 1(0.85) |
| Myalgia | 0 | 1(2.94) | 0 | 0 | 0 | 1(0.85) |
| Neck pain | 0 | 1(2.94) | 0 | 0 | 0 | 1(0.85) |
| Rheumatoid arthritis | 0 | 0 | 1(1.19) | 0 | 1(0.85) | 0 |
| Scoliosis | 0 | 0 | 0 | 1(1.19) | 0 | 1(0.85) |
| **Gastrointestinal disorders** | 4(11.76) | 0 | 0 | 2(2.38) | 4(3.39) | 2(1.69) |
| Abdominal pain upper | 2(5.88) | 0 | 0 | 0 | 2(1.69) | 0 |
| Abdominal discomfort | 0 | 0 | 0 | 1(1.19) | 0 | 1(0.85) |
| Abdominal pain | 1(2.94) | 0 | 0 | 0 | 1(0.85) | 0 |
| Abdominal pain lower | 1(2.94) | 0 | 0 | 0 | 1(0.85) | 0 |
| Gastric polyps | 0 | 0 | 0 | 1(1.19) | 0 | 1(0.85) |
| **Nervous system disorders** | 1(2.94) | 1(2.94) | 0 | 1(1.19) | 1(0.85) | 2(1.69) |
| Headache | 1(2.94) | 0 | 0 | 1(1.19) | 1(0.85) | 1(0.85) |
| Lethargy | 0 | 1(2.94) | 0 | 0 | 0 | 1(0.85) |
| **Respiratory, thoracic and mediastinal disorders** | 0 | 0 | 0 | 3(3.57) | 0 | 3(2.54) |
| Cough | 0 | 0 | 0 | 2(2.38) | 0 | 2(1.69) |
| Rhinitis allergic | 0 | 0 | 0 | 1(1.19) | 0 | 1(0.85) |
| **Metabolism and nutrition disorders** | 0 | 0 | 0 | 2(2.38) | 0 | 2(1.69) |
| Dyslipidaemia | 0 | 0 | 0 | 1(1.19) | 0 | 1(0.85) |
| Hypercholesterolaemia | 0 | 0 | 0 | 1(1.19) | 0 | 1(0.85) |
| **Skin and subcutaneous tissue disorders** | 0 | 0 | 0 | 2(2.38) | 0 | 2(1.69) |
| Alopecia | 0 | 0 | 0 | 1(1.19) | 0 | 1(0.85) |
| Rash | 0 | 0 | 0 | 1(1.19) | 0 | 1(0.85) |
| **Blood and lymphatic system disorders** | 0 | 0 | 1(1.19) | 0 | 1(0.85) | 0 |
| Lymphadenopathy | 0 | 0 | 1(1.19) | 0 | 1(0.85) | 0 |
| **Injury, poisoning and procedural complications** | 0 | 0 | 0 | 1(1.19) | 0 | 1(0.85) |
| Hand fracture | 0 | 0 | 0 | 1(1.19) | 0 | 1(0.85) |
| **Psychiatric disorders** | 0 | 1(2.94) | 0 | 0 | 0 | 1(0.85) |
| Insomnia | 0 | 1(2.94) | 0 | 0 | 0 | 1(0.85) |
| **Surgical and medical procedures** | 0 | 0 | 0 | 1(1.19) | 0 | 1(0.85) |
| Haemorrhoid operation | 0 | 0 | 0 | 1(1.19) | 0 | 1(0.85) |

TEAEs = treatment-emergent adverse events, SOC = system organ class, PT = preferred term

Note: Denominator of percentage is the number of subjects in the each treatment group.

TEAEs are displayed as number of subjects(percentage of subjects).

Where one subject experiences the same preferred term more than once, the preferred term is counted only once in the most relationship category.
